# Structural Elucidation and Covalent Modulation of the Autorepressed Orphan Nuclear Receptor NR2F6

**DOI:** 10.1021/acschembio.5c00475

**Published:** 2025-09-10

**Authors:** Guido J. M. Oerlemans, Maxime C. M. van den Oetelaar, Siebe P. van den Elzen, Luc Brunsveld

**Affiliations:** Laboratory of Chemical Biology, Department of Biomedical Engineering and Institute of Complex Molecular Systems, 3169 Technische Universiteit Eindhoven, 5612 AZ Eindhoven, The Netherlands

## Abstract

The orphan nuclear receptor NR2F6 (Nuclear Receptor subfamily 2 group F member 6) is an emerging therapeutic target for cancer immunotherapy. Upregulation of NR2F6 expression in tumor cells has been linked to proliferation and metastasis, while in immune cells NR2F6 inhibits antitumor T-cell responses. Small molecule modulation of NR2F6 activity might therefore be a novel strategy in cancer treatment, benefiting from this dual role of NR2F6. However, there are no molecular strategies available for targeting NR2F6, hampered among others by lack of structural insights and appropriate biochemical assays. To overcome these challenges, several noncanonical nuclear receptor coregulator peptide motifs were identified to be constitutively recruited to the NR2F6 ligand binding domain (LBD). Co-crystallization of the NR2F6 LBD with a peptide from the coregulator Nuclear Receptor Binding SET Domain Protein 1 (NSD1) enabled, for the first time, the structural elucidation of the unliganded (apo) form of NR2F6. This revealed an autorepressed, homodimeric LBD conformation in which helix 12 folds over the canonical coregulator binding site, generating an alternative contact surface for NSD1 binding. Screening of a focused library of covalent NR probes identified compounds that preferentially target a cysteine residue near the NSD1 binding site, inhibiting NR2F6 coregulator recruitment. Combined, these results provide structural insights into the ligand-independent transcriptional activity of NR2F6 and may serve as a starting point for the development of novel NR2F6 modulators.

## Graphical abstract

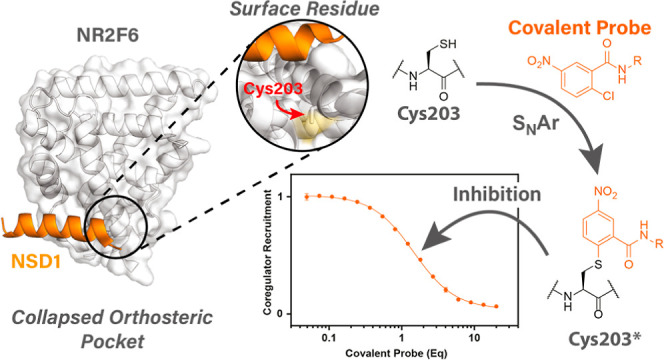

## Introduction

Nuclear receptors (NRs) are a family of ligand-inducible transcription factors that regulate gene expression through the recruitment of coregulator proteins to their hormone response elements on the DNA.^1–3^ Dysregulation of NR signaling can result in metabolic disorders, neoplasia and inflammatory diseases.^1,4^ The 48 human members of the NR superfamily can be divided in three classes based on the discovery of their natural ligands; the classic endocrine hormone receptors, the adopted orphan receptors and the true orphan receptors for which no endogenous ligands have been identified up to date.^5^ Medicinal chemistry efforts focusing on the first two classes of NRs have been highly successful in the clinic, with approximately 16% of all the FDA-approved drugs targeting these NRs.^6^ The remaining true orphan NRs hold immense, but underexplored therapeutic potential.^7^

NR2F6 (Nuclear Receptor subfamily 2 group F member 6), also known as V-erbA-related protein 2 (EAR-2) or chicken ovalbumin upstream promoter-transcription factor 3 (COUP-TF3), is an orphan NR of which the biological role has only recently become apparent,^8,9^ despite its relatively early discovery.^10,11^ In effector CD4^+^ and CD8^+^ T cells, NR2F6 represses the expression of interleukin-2 (IL-2), interferon gamma (IFN-γ) and tumor necrosis factor alpha (TNFα), limiting T cell activation within the tumor microenvironment.^8,12^ Knockout of NR2F6 in mice increases cytokine expression by tumor-infiltrating T cells, improving antitumor responses and reducing metastasis.^8^ Moreover, partial or complete knockout of NR2F6 synergizes with immune checkpoint blockade therapy.^13,14^ High NR2F6 expression in tumor cells is linked to proliferation, therapy resistance and poor prognosis in several solid cancers.^15^ This dual pro-tumor activity of NR2F6 in both immune and cancer cells makes modulation of NR2F6 a promising strategy for cancer therapy.^15,16^

As a true orphan receptor, endogenous ligands modulating NR2F6 activity remain unknown.^15^ Studies on the closely related NR2F family members COUP-TFI (NR2F1) and COUP-TFII (NR2F2) show that these NRs can be modulated through small molecules that target their ligand binding domain (LBD). A metabolite screening identified 1-deoxysphingolipids^17^ as binders of the NR2F1/NR2F2 LBD, promoting NR2F1/NR2F2 transcriptional activity. Screening efforts identified CIA1/CIA2 ^18^ and 4-methoxynaphthol^19^ as inhibitors targeting the LBD of NR2F2, while C-DIMs^20^ have been reported as LBD-targeting activators of NR2F1. A recent cellular screening campaign identified potential small molecule NR2F6 modulators, albeit with only weak effects on the NR2F6 LBD in vitro.^21^ Beyond the NR2F family, ligand development efforts have been successful for the related orphan receptor TLX (NR2E1).^22,23^ However, the absence of structural information and appropriate biochemical assays, a common challenge in the field of NR2 receptors,^24^ hampers the development of NR2F6 modulators.

In this study, therefore, the cofactor binding profile of the NR2F6 LBD was characterized, identifying several noncanonical coregulator peptides as constitutive binders of the NR2F6 LBD. Using a maltose-binding protein (MBP) fusion construct, the first crystal structure of the apo NR2F6 LBD was elucidated, in complex with a coregulator peptide derived from Nuclear Receptor Binding SET Domain Protein 1 (NSD1). The crystal structure revealed that the apo LBD is in a homodimeric, autorepressed conformation in which helix 12 (H12, AF-2) folds over the canonical coregulator binding site, preventing the binding of canonical LXXLL/LXXXIXXXL coregulator motifs. Based on this structural data, cysteine residue 203 (C203) was identified as a potential entry point for NR2F6 modulation. Screening of a covalent probe library revealed that this residue can be preferentially targeted, and covalent engagement of C203 inhibited the recruitment of coregulators to the NR2F6 LBD, providing potential starting points for the development of NR2F6 modulators.

## Results and Discussion

Although NR2F6 has been demonstrated to exert both activating^25–27^ and repressing^9,28–30^ effects on gene transcription, the cofactor proteins mediating NR2F6 transcriptional activity remain to be elucidated. It has been proposed that the mechanism behind NR2F6 transcriptional repression is the direct recruitment of corepressor proteins to DNA-bound NR2F6 homo- or heterodimers (e.g., with RXRs),^15,31^ while transcriptional activation could occur through direct recruitment of coactivator proteins to DNA-bound NR2F6 ^26^ or through indirect recruitment via the interaction with other DNA-bound transcription factors.^15,31^ Since the NR2F6 LBD itself confers transcriptional repressive activity,^32^ the direct recruitment of NR coregulators to the NR2F6 LBD was investigated (Figure 1A).

**Figure 1:**
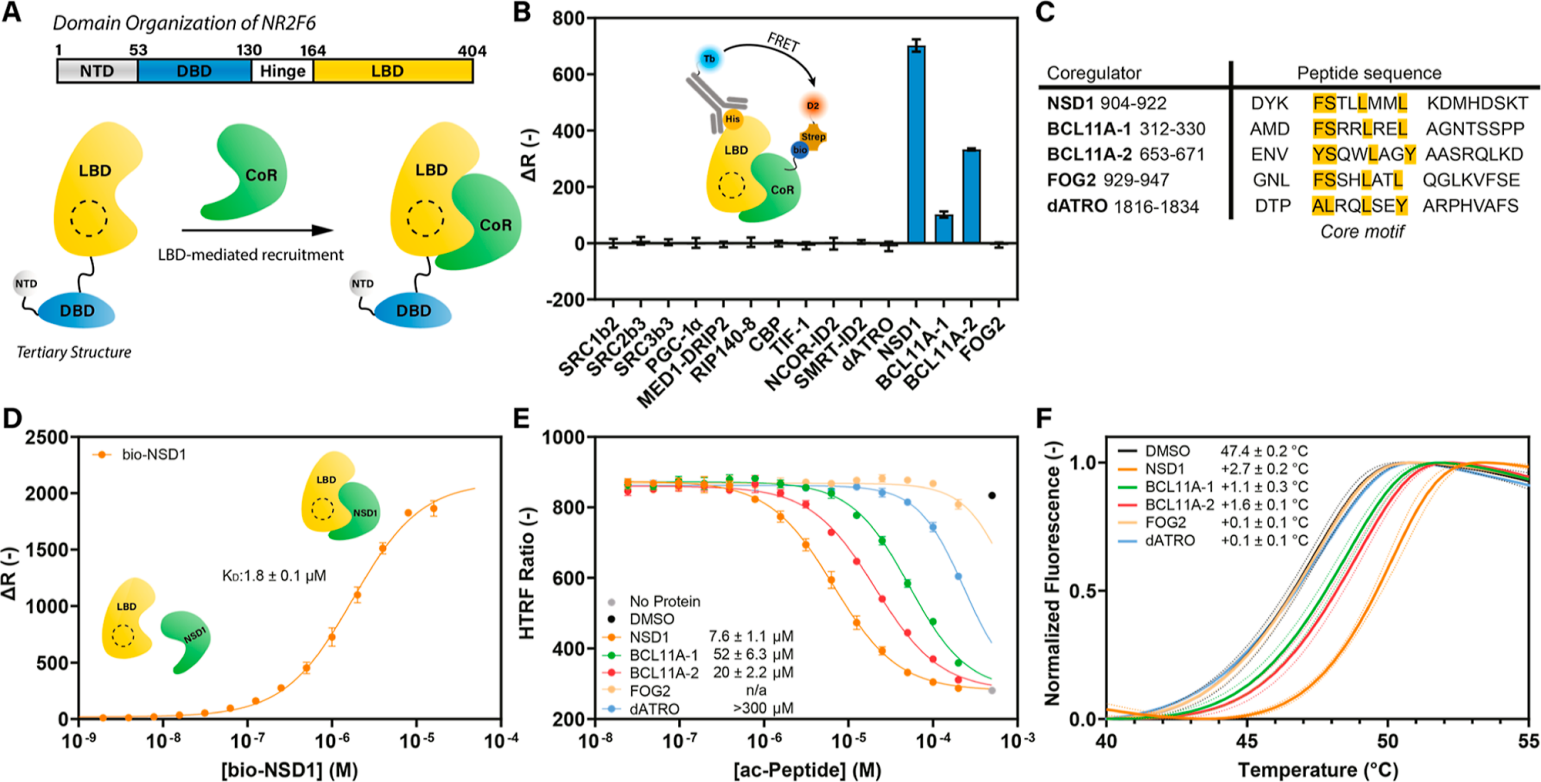
Cofactor profiling of the NR2F6 LBD. (A) Domain organization of NR2F6 and concept of LBD-mediated recruitment of coregulator proteins. (B) TR-FRET cofactor profiling of NR2F6 LBD. Data recorded in triplicate. (C) Sequence alignment of screened noncanonical NR coregulator peptides. (D) TR-FRET concentration–response curve of bio-NSD1 binding to His-NR2F6 LBD. Data shown is the average and standard deviation of three independent experiments. (E) TR-FRET concentration–response curves of acetylated NSD1, BCL11A-1, BCL11A-2, FOG2 and dATRO in a bio-NSD1 displacement assay. Data were recorded in triplicate; data shown is representative of three independent experiments. Reported values represent the average and standard deviation of three independent experiments. (F) DSF curves of apo NR2F6 LBD in the presence of DMSO or NSD1, BCL11A-1, BCL11A-2, FOG2 or dATRO peptide. Data shown is the average and standard deviation of three independent experiments.

### Recruitment of F/YSXXLXXL/Y Motifs by the NR2F6 LBD

A library of biotinylated NR coregulator motifs (sequences listed in Table S1) was screened for NR2F6 LBD binding in a single-point time-resolved fluorescence resonance energy transfer (TR-FRET) assay. Canonical LXXLL coactivator motifs and LXXXIXXXL corepressor motifs showed no binding to apo NR2F6 LBD (Figure 1B). Therefore, noncanonical NR coregulator motifs were evaluated including the Atro box peptide derived from the *Drosophila* homologue of the TLX corepressor Atrophin^33^ (dATRO), as well as several F/YSXXLXXL/Y motifs previously reported or proposed to selectively interact with the NR2E/F families^34^ (Figure 1C). In line with previous work by Chan et al.,^34^ two F/YSXXLXXL/Y peptide motifs derived from BCL11A, a corepressor of the NR2F family^35^ and TLX NR,^36^ were constitutively recruited to the NR2F6 LBD (Figure 1B). The BCL11A-2 peptide (also known as BCL11A-RID2) exhibited stronger binding than the BCL11A-1 peptide (also known as BCL11A-RID1), as evidenced by a higher TR-FRET ratio. An FSXXLXXL peptide derived from the bifunctional coregulator NSD1, which can act both as coactivator and corepressor for various NRs,^37^ demonstrated stronger recruitment than both BCL11A-derived peptides. Although previously proposed to directly mediate binding to the NR2F6 LBD,^34^ an FSXXLXXL peptide derived from the known NR2F6 corepressor FOG2 ^38^ was not recruited to the NR2F6 LBD in the primary coregulator screen. Similarly, the dATRO peptide showed no recruitment.

The biotinylated NSD1 peptide (bio-NSD1) demonstrated concentration-dependent binding to the NR2F6 LBD with a K_D_ of 1.8 ± 0.1 μM (Figure 1D). This enabled the development of a bio-NSD1 displacement TR-FRET assay using unlabeled counterparts of the noncanonical coregulator motifs, allowing for a quantitative comparison. Acetylated NSD1 (ac-NSD1) displaced bio-NSD1 most potently with an IC_50_ of 7.6 ± 1.1 μM (Figures 1E and S1). The ac-BCL11A-1 and ac-BCL11A-2 peptides displaced bio-NSD1 with larger IC_50_ values of 52 ± 6.3 μM and 20 ± 2.2 μM, respectively, in line with the primary coregulator profiling. The competition with NSD1 suggests that all three peptides compete for a similar binding site. Very weak displacement was observed for ac-dATRO, with an extrapolated IC_50_ exceeding 300 μM. No significant displacement was observed for ac-FOG2. Differential scanning fluorimetry (DSF) experiments (Figure 1F) aligned with the TR-FRET data. The apo NR2F6 LBD has a melting temperature of 47.4 ± 0.2 °C. The ac-NSD1 peptide induced NR2F6 LBD thermal stabilization with a Δ*T*_m_ of 2.7 ± 0.2 °C. The ac-BCL11A-1 and ac-BCL11A-2 peptides showed weaker stabilization with a Δ*T*_m_ of 1.1 ± 0.3 °C and Δ*T*_m_ of 1.6 ± 0.1 °C, respectively. Both ac-dATRO and ac-FOG2 did not affect the melting temperature of the NR2F6 LBD.

### Co-Crystal Structure of the NR2F6/NSD1 Complex

NR2F6 remains among the few of the 48 human nuclear receptor LBDs to be crystallized, likely owing to the unstable, aggregation-prone nature of the purified NR2F6 LBD. Inspired by Xu and colleagues, who used an MBP fusion strategy to crystallize the SHP,^39^ PNR^40^ and TLX^33^ orphan NRs, a similar approach was followed to improve NR2F6 stability. Sequence alignment of the NR2F6 LBD with the related autorepressed receptors COUP-TF2, TLX and PNR (Figure S2A) revealed that the NR2F6 LBD likely possesses a relatively short helix 1 (H1), followed by a flexible alanine/glycine repeat leading up to H3. Additionally, the LBD contains a relatively short but disordered stretch of amino acids following H12 at the C-terminus. To improve protein stability, the NR2F6 LBD was truncated to residues 199–393 and fused to a mutant MBP containing surface entropy reduction (SER) mutations^41,42^ (Figure S2B). Although this construct improved protein stability significantly, enabling protein concentrations suitable for crystallography, initial crystallization attempts were unsuccessful.

Incubation of the MBP-NR2F6 fusion construct with the BCL11A-1, BCL11A-2, or NSD1 peptide increased the protein melting temperature (Figure S2C), likely improving the conformational homogeneity of the NR2F6 LBD. Initial needle-like crystals were obtained by preincubation of MBP-NR2F6 with the NSD1 peptide. Subsequent additive screening optimized the initial hit condition, yielding diffraction-grade crystals that enabled elucidation of the NR2F6 LBD/NSD1 peptide cocrystal structure at 2.6 Å in the P6_1_ space group (crystal data collection and refinement statistics are listed in Table S2).

The asymmetric unit (Figure S3A) contains two copies of the MBP-NR2F6 fusion protein (termed monomer A and B) and two copies of the NSD1 peptide. Both copies of the NR2F6 LBD have a highly similar three-dimensional structure and overlay closely with an RMSD of 0.191 Å (Figure S3B). NR2F6 forms a typical NR homodimer, with the dimerization interface formed by H7, H9 and H10 and the loops between H8–H9 and H9–H10 (Figure 2A), similar to previously reported crystal structures of NR homo- and heterodimers. Analytical SEC experiments showed MBP–NR2F6 to also, and exclusively, form homodimers in solution (Figure S4), while the appended MBP is monomeric in solution, irrespective of SER mutations.^43^ In both monomers of NR2F6, H10 breaks at P364, with the lower half of this helix collapsing into the orthosteric ligand binding pocket. As a result, the orthosteric pocket is densely packed with hydrophobic and aromatic side chains from H3, H5, H7 and H10 and the loop between H5 and H7 (see also Figure 4B). The collapsed orthosteric ligand binding pocket allows H12 to fold over the canonical coregulator binding site (Figure 2A,D), binding the interface formed by H3, H4, H5 and H10 with the hydrophobic sequence IETLIRDML (underlined residues face the interaction surface, Figure S3C). The positioning of H12 is further stabilized by hydrogen bonds between W239 of H4 and E383 of H12, and R218 of H3 and the backbone of M389 of H12. The binding mode of H12 is reminiscent of the binding of LXXXIXXXL corepressor motifs to NRs. The overall autorepressed structure of the NR2F6 LBD is similar to that of the previously crystallized autorepressed orphan receptors, including COUP-TF2 (RMSD of 0.685 Å) and TLX (RMSD of 0.693 Å) (Figure S3D). In addition to the typical NR dimerization interface, the removal of the predicted H1 of NR2F6 in the crystallography construct facilitates a second dimerization interface on the opposite site of the receptor formed by H3, H8, H12 and the NSD1 peptide (Figure S3E). Both dimerization sites result in the formation of a NR2F6 pentamer (Figure S3F). As the full-length NR2F6 LBD is predicted to contain H1 (Figure S2A), this second dimerization interface is likely induced by crystal packing of the engineered MBP-NR2F6 fusion protein and is not relevant to NR2F6 function in a full-length LBD context.

**Figure 2:**
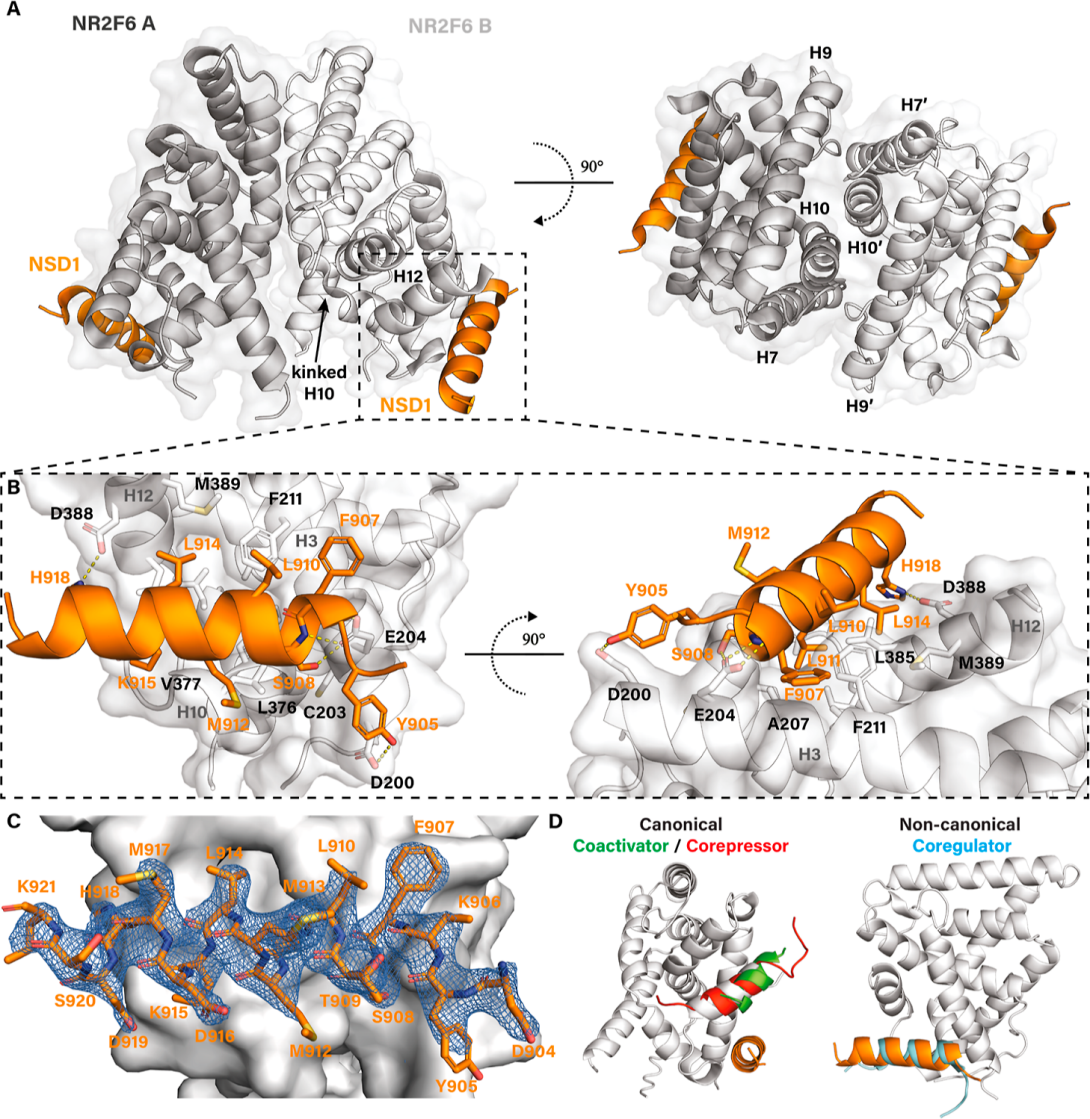
Co-crystal structure of NR2F6 with NSD1 peptide. (A) NR2F6 LBD homodimer (dark and light gray) in complex with NSD1 peptide (orange). (B) Zoom-in of the NR2F6/NSD1 interaction of monomer B. (C) 2Fo-Fc electron density map (blue mesh) of NSD1 of monomer B contoured at 1.0 σ. (D) Overlay of the NR2F6/NSD1 complex with the canonical SRC-1 coactivator peptide from an agonist bound RXRα structure (green, PDB: 6JNR) and the SMRT corepressor peptide from an antagonist bound PPARα structure (red, PDB: 1KKQ) or the noncanonical dAtrophin peptide from the apo TLX structure (light blue, PDB: 4XAJ).

**Figure 3:**
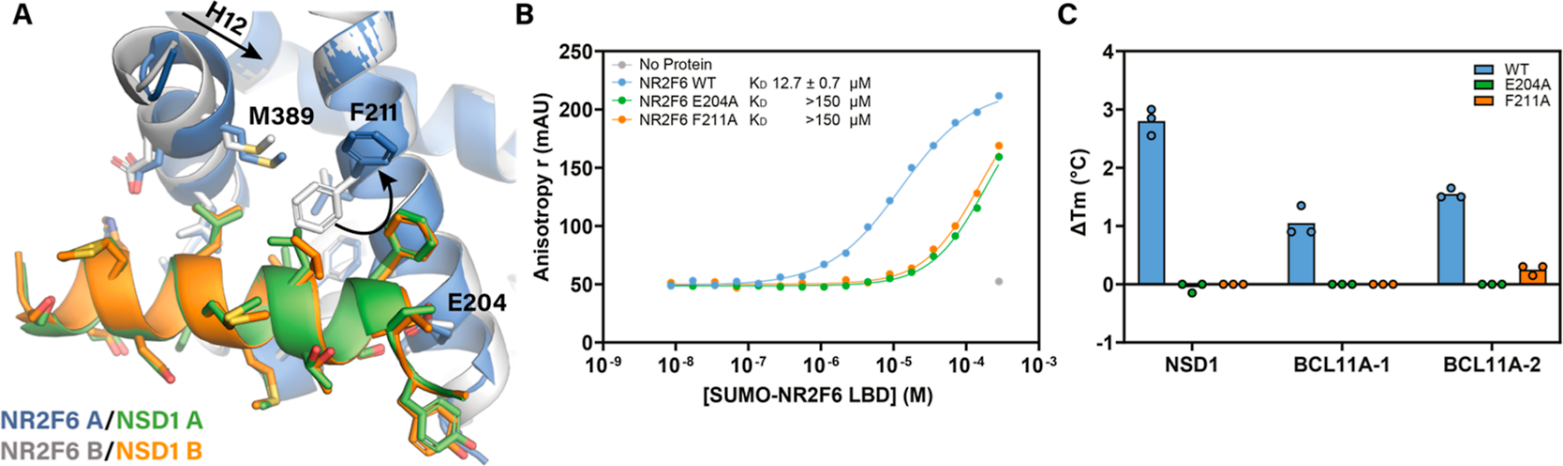
Functional analysis of the NR2F6/NSD1 interaction. (A) Structural overlay of both NR2F6/NSD1 copies present in the asymmetric unit. (B) FA concentration–response curves of FAM-NSD1 recruitment by wildtype NR2F6, NR2F6^E204A^ and NR2F6^F211A^. Data shown is the average and standard deviation of three independent experiments. (C) Thermal stabilization of NR2F6, NR2F6^E204A^ and NR2F6^F211A^ by NSD1, BCL11A-1, and BCL11A-2 coregulator peptides. Data shown is the average and standard deviation of three independent experiments.

**Figure 4:**
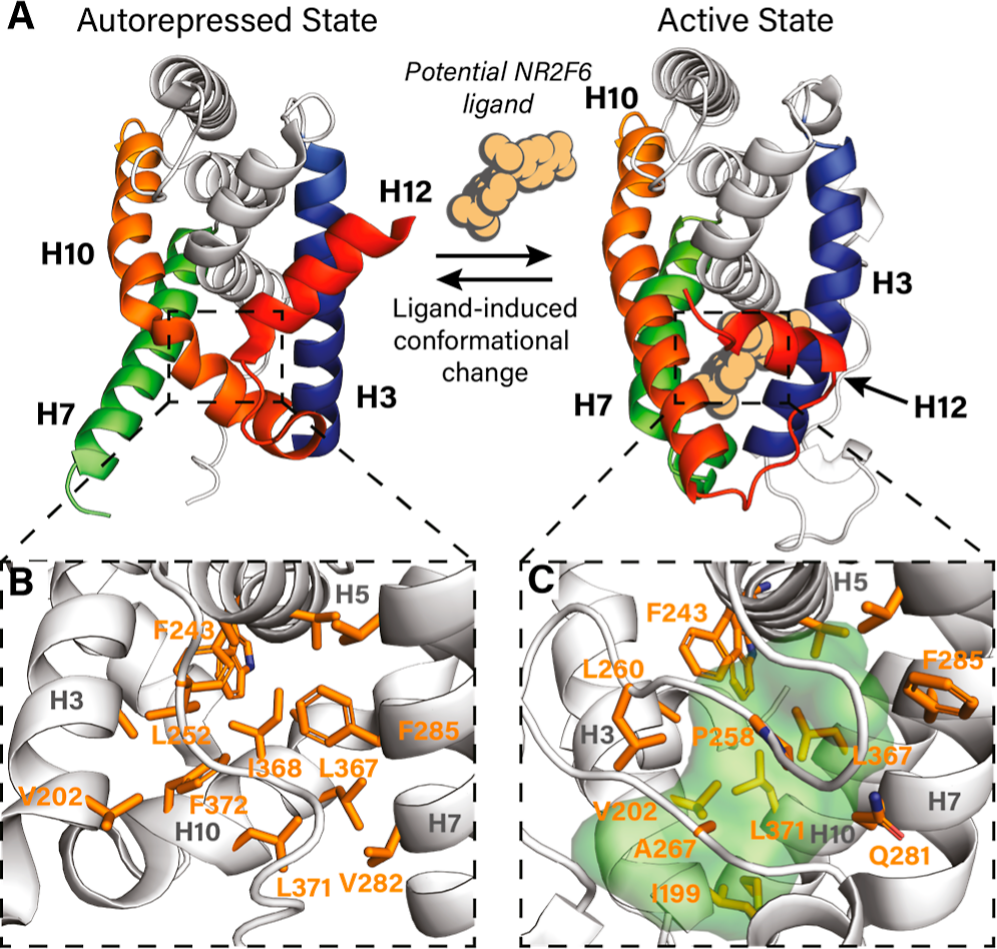
Prediction of the NR2F6 orthosteric pocket. (A) Conformational change of the NR2F6 LBD upon hypothetical ligand binding. (B) Zoom-in of the orthosteric pocket of the crystallized autorepressed state of NR2F6, with H10 folding into the canonical ligand binding pocket. (C) Zoom-in of the orthosteric pocket of the modeled active state of NR2F6. The predicted volume available for ligand binding is shown in green. Residues facing the protein interior are shown as orange sticks. Labeled residues undergo major reorientation between the two protein conformations.

Both copies of the NR2F6/NSD1 interaction are highly similar (Figures 2B and S3H). In both cases, full density is observed for the NSD1 peptide, except for the C-terminal T922 residue (Figures 2C and S3G). The α-helical NSD1 peptide binds the interface formed by H3, H10 and H12, with L911 and L914 of NSD1 buried into the hydrophobic surface pocket created by residues A207, L210 and F211 on H3, F373, L376 and V377 on H10 and T380, I382, L385 and M389 on H12 (Figure 2B for monomer B, Figure S3H for monomer A). The hydrophobic NSD1 residues F907, L910, M912 and H918 engage in additional hydrophobic contacts with the NR2F6 surface. The interaction is stabilized by polar interactions on both sides of the peptide. On the N-terminal side of NSD1, E204 on H3 forms three hydrogen bonds with the NSD1 peptide, one with the side chain of S908 and two with the backbone nitrogen atoms of F907 and S908. D200 of H3 forms a hydrogen bond with the side chain of NSD1 Y905. At the peptide C-terminus, the side chain of H918 of NSD1 forms a hydrogen bond with D388 of H12 (Figure 2B). The polar interactions between NSD1 and NR2F6 are slightly different in monomer A (Figure S3H). Y905 of NSD1 forms two hydrogenbonds with the backbone of I199 and the side chain of N201 of NR2F6, whereas the hydrogen bond between D388 and H918 is disrupted. These differences are likely induced by crystal packing. The MBP of monomer A is fused to NR2F6 via a solid helix compared to a flexible loop in monomer B, imposing a different orientation of the most N-terminal residues of NR2F6. In addition, H12 of monomer A is slightly displaced due to a crystal packing movement of F211 (vide infra), disrupting the hydrogen bonding interaction between D388 and H918. As monomer B is free of these crystallographic artifacts, the NR2F6/NSD1 binding mode of monomer B (as illustrated in Figure 2B) is considered to best resemble the actual NR2F6/NSD1 interaction. Structural overlay with a canonical coactivator motif (SRC-1 from an agonist bound RXRα structure) and a canonical corepressor motif (SMRT from an antagonist bound PPARα structure) highlights H12 sterically clashing with their recruitment (Figure 2D), explaining the lack of canonical coregulator recruitment observed in the TR-FRET screen. The noncanonical dAtrophin peptide from the Atrophin/TLX structure^33^ overlays well with the NSD1 peptide (Figure 2D), reinforcing the hypothesis that the recruitment of noncanonical coregulators to the autorepressed LBD is a conserved structural mechanism for gene regulation by repressive orphan nuclear receptors.^33^

### Validation Analysis of the NR2F6/NSD1 Interaction

Overlay of both NR2F6/NSD1 complexes, present within the asymmetric unit of the crystal structure, reveals a key difference between both monomers. The F211 residue of NR2F6, which engages in close hydrophobic contacts with the NSD1 peptide in monomer B, faces away from the NSD1 peptide in monomer A (Figure 3A). This opens up the tight hydrophobic surface pocket bound by NSD1 residues L911 and L914, facilitating the movement of H12 toward H3 and disrupting the hydrogen bond between D388 of NR2F6 and H918 of NSD1. To validate the NR2F6/NSD1 interaction as observed in the crystal structure and to probe the subtle difference between both monomers in more detail, we engineered the NR2F6^E204A^ and NR2F6^F211A^ mutants. In fluorescence anisotropy (FA) experiments (Figure 3B), wildtype NR2F6 LBD recruited FAM-labeled NSD1 in a concentration-dependent manner with a *K*_D_ of 12.7 ± 0.7 μM, which is in line with the binding affinity of the bio-NSD1 peptide in the TR-FRET assay. Both the NR2F6^E204A^ and NR2F6^F211A^ mutant exhibited more than 10-fold lower affinity for NSD1 with an extrapolated *K*_D_ > 150 μM. Both mutants displayed melting temperatures comparable to wildtype LBD in DSF studies (46.4 °C for NR2F6^E204A^, 47.2 °C for NR2F6^F211A^ and 47.4 °C for wildtype NR2F6, Figure S5A), excluding instability of the mutant proteins as the cause of the reduced NSD1 binding affinity.

The loss of NSD1 binding was further corroborated by DSF experiments (Figure 3C), as the stabilizing effect of the NSD1, BCL11A-1 or BCL11A-2 coregulator peptides on NR2F6 unfolding was lost upon introduction of either the E204A or F211A mutation. Moreover, NR2F6^E204A^ and NR2F6^F211A^ failed to induce recruitment of these coregulator peptides in TR-FRET experiments (Figure S5B). Combined, these results verify the observed binding mode of NSD1 in the crystal structure and confirm that the NR2F6 LBD is in a collapsed conformation in solution when binding the NSD1 peptide. Furthermore, the loss of NSD1 binding by the NR2F6^F211A^ mutant substantiates the hypothesis that the movement of F211 away from the NSD1 peptide observed in monomer A is caused by crystal packing contacts on the artificial dimer interface (zoom-in Figure S3E), confirming the interaction as observed in monomer B (Figure 2B) most representative of the native NR2F6/NSD1 interaction.

The BCL11A-1 and BCL11A-2 F/YSXXLXXL/Y motifs contain a methionine/asparagine at the corresponding Y905 position of NSD1 and a threonine/arginine at the H918 position toward the C-terminus of NSD1 (Figure 1C). These BCL11A residues are more flexible, have less hydrophobic bulk or lack the capability of forming hydrogen bonds, thus potentially contributing to the decreased affinity of BCL11A-1 and BCL11A-2 for NR2F6 compared to NSD1. The NR2F6/NSD1 cocrystal structure does not provide a structural explanation for the lack of binding of the FOG-2 peptide, as no obvious steric clashes with the LBD are anticipated based on the peptide sequence. The lack of FOG2 peptide binding is possibly due to the lack of α-helicity of the peptide. This is in line with structural predictions by AlphaFold, as the FSXXLXXL sequence is predicted to be disordered in the full-length FOG-2 protein, whereas the F/YSXXLXXL/Y motifs of the full-length BCL11A and NSD1 proteins are predicted to be α-helical domains.^44,45^ Although beyond the scope of this work, α-helicity studies^46^ and alanine scanning experiments^47^ with the different F/YSXXLXXL/Y peptides could provide more information on the observed differences in binding affinity for NR2F6.

### Probing the Active Conformation of the NR2F6 LBD

Several potential modulators of NR2F6 transcriptional activity, targeting the NR2F6 LBD, have been described in literature, including the known NR modulators troglitazone^21,48^ and 9-cis-retinoic acid^32^ (Figure S6A). Despite the collapsed apo structure of NR2F6 in the crystal structure, NR LBDs alternate between different conformations in solution and ligand binding can shift this dynamic conformational equilibrium.^49^ To probe the potential liganded conformation of the NR2F6 LBD, a homology model was generated based on the 9-cis-retinoic acid-bound conformation of RXRα (Figure 4A). According to this model structure, straightening of the collapsed H10 and refolding of H12 upon ligand binding could open up the collapsed LBD of NR2F6 (Figure 4B), generating a sizable, hydrophobic ligand binding pocket of approximately 800–900 Å^3^ (Figure 4C). This pocket would be large enough to accommodate small molecules. Ligand binding to the orthosteric pocket of NR2F6 could potentially induce displacement of the F/YSXXLXXL/Y motifs or even facilitate the recruitment of canonical LXXLL motifs. Although an orthosteric ligand binding pocket might thus exist within the NR2F6 LBD, both troglitazone and 9-cis-retinoic acid could not be validated as modulators of the NR2F6 LBD as both failed to significantly alter recruitment of F/YSXXLXXL/Y motifs or induce recruitment of canonical NR coregulators (Figure S6B). This highlights the need for novel chemical matter to modulate NR2F6 activity through its LBD.

### Discovery of Covalent NR2F6 Modulators

The NR2F6/NSD1 cocrystal structure reveals that C203 of NR2F6 is located close to the NSD1 binding site (Figure 5A). Moreover, C203 is hypothesized to face the interior of the ligand binding pocket in the modeled active conformation of NR2F6 (Figure 5A). We therefore hypothesized that this cysteine could potentially be preferentially targeted over the other two cysteine residues (C304 and C316) in the NR2F6 LBD (Figure S7A), providing an opportunity for covalent modulation of NR2F6 activity. Therefore, we screened a covalent probe library based on various electron-deficient haloarenes.^50^ This type of warhead has previously been successfully employed to target similarly located cysteine residues in the nuclear receptors PPARγ,^49,51–53^ PPARδ^54^ and RORγt.^55^ Covalent attachment of this warhead is based on a nucleophilic aromatic substitution reaction (S_N_Ar) in which the nucleophilic thiol side chain of a cysteine residue attacks the warhead, displacing the chlorine atom (Figure S7B).

**Figure 5:**
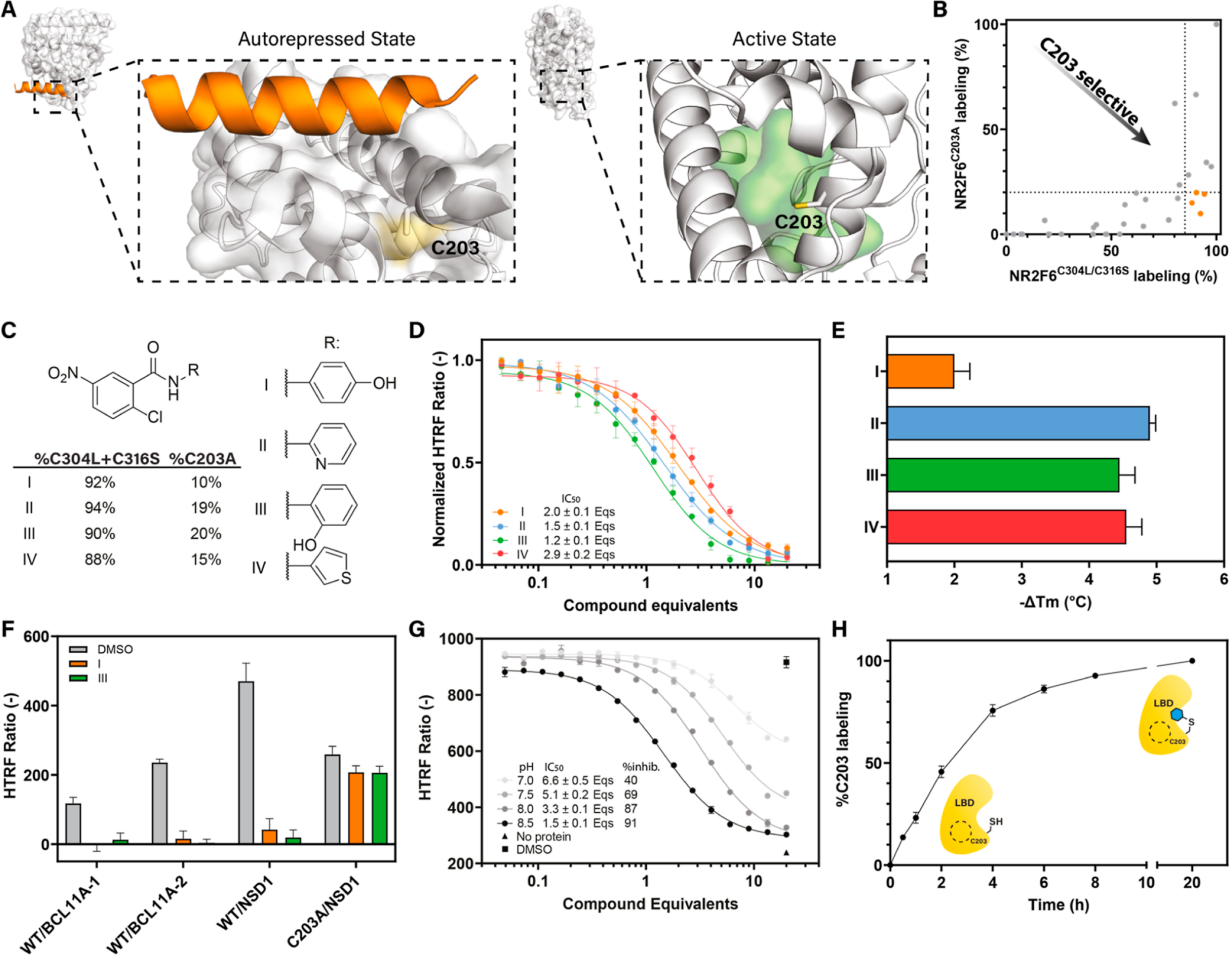
Covalent modulation of NR2F6 activity. (A) Zoom-in of the crystallized autorepressed and modeled active state of the NR2F6 LBD highlighting the C203 residue. C203 is surface accessible in the autorepressed conformation and faces into the ligand binding pocket in the predicted active conformation. (B) MS-based library screening of NR2F6^C203A^ and NR2F6^C304L/C316S^ LBD. Hit selection threshold of each screen is shown as a black dashed line. Gray dots represent individual library members. Orange dots represent compounds **I–IV**. (C) Chemical structure and MS-screening labeling results of compound **I–IV**. (D) Dose–response curves of **I–IV** in the bio-NSD1 TR-FRET displacement assay. Data shown is the average and standard deviation of three independent experiments. (E) Effect of **I–IV** on the NR2F6 LBD melting temperature in DSF. Data shown is the average and standard deviation of three independent experiments. (F) Effect of **I** and **III** on BCL11A-1, BCL11A-2 and NSD1 recruitment to wildtype NR2F6 LBD and NR2F6^C203A^ LBD in TR-FRET. Data shown is the average and standard deviation of three independent experiments. (G) pH-dependent displacement of bio-NSD1 in TR-FRET by **I**. Data recorded in triplicate; data, average and standard deviation shown is representative of two independent experiments. (H) Q-ToF MS time-dependent labeling of NR2F6^C304L/C316S^ LBD by **I**. Data shown is the average and standard deviation of three independent experiments.

To screen for C203-selective compounds, two different cysteine mutants of the NR2F6 LBD were designed. The NR2F6^C304L/C316S^ double mutant was used to profile probe binding to C203. The double mutant was designed based on structural alignment with other NRs, demonstrating that an (iso)­leucine residue is common at the C304 position. Furthermore, C316 is located on a solvent exposed flexible loop and was therefore mutated into a serine. To screen for off-target probe binding, the NR2F6^C203A^ mutant was used which lacks the target cysteine. The stability of both cysteine mutants was investigated via DSF (Figure S7C). The melting temperature of NR2F6^C203A^ was reduced compared to the wildtype LBD (Δ*T*_m_ of −2.4 °C), possibly owing to a disruption of the tight packing of the collapsed state of the receptor by the smaller alanine residue. NR2F6^C304L/C316S^ showed comparable stability to wildtype LBD (Δ*T*_m_ of 0.9 °C). The binding of the covalent probes to both cysteine mutants was profiled in a mass-spectrometry (MS) based setup (Figures 5B, and S7D–F). The screening results of the full library are listed in Table S3. To select hits for further investigation, a cutoff of ≥85% C203 labeling and ≤20% C304/C316 labeling was chosen, resulting in the identification of compounds **I–IV** (Figure 5C), which are all based around the 2-chloro-5-nitrobenzamide warhead.

Titration of compounds **I–IV** in the TR-FRET NSD1 recruitment assay revealed that all four compounds inhibited the recruitment of the bio-NSD1 peptide in a dose-dependent manner with comparable potencies (Figure 5D), requiring 1.2 to 2.9 equivalents of compounds (with respect to NR2F6) to achieve 50% inhibition of NSD1 recruitment. DSF experiments (Figure 5E) of covalently bound NR2F6^C304L/C316S^ showed that covalent engagement of C203 influences the NR2F6 LBD conformation, inducing a destabilization of the protein. On average, **I** destabilized the NR2F6 LBD by 2.0 °C, while **II–IV** destabilized the melting temperature more strongly with a 4.5 to 4.9 °C decrease. Destabilization upon ligand binding has been observed before for the autorepressed TLX receptor, and might be attributed to the opening of the collapsed (stable) conformation of the LBD, releasing H12 from the AF-2 site.^56^ The significant difference in Δ*T*_m_ for **I** compared to **II–IV**, despite minor chemical modification, illustrates the potential of these probes to be developed into NR2F6 stabilizing ligands. To confirm the generalizability of inhibition of coregulator recruitment, the effects of **I** and **III** on the recruitment of NSD1, BCL11A-1, and BCL11A-2 was investigated in TR-FRET (Figure 5F). Both compounds strongly inhibited the recruitment of BCL11A-1 (complete inhibition for **I** and a 9-fold reduction in recruitment for **III**, relative to DMSO), BCL11A-2 (15-fold reduction in recruitment for **I** and complete inhibition for **III**, relative to DMSO), and NSD1 (11-fold reduction in recruitment for **I** and 24-fold reduction in recruitment for **III**, relative to DMSO), upon full ligation to the NR2F6 LBD. Following mutation of the C203 residue to alanine, the compounds lost almost all of their inhibitory activity in the TR-FRET assay (Figure 5F, 1.2-fold reduction in recruitment relative to DMSO), confirming the selective and covalent targeting of C203 by both compounds.

Compound **I** was subsequently evaluated in more detail, being the most C203-selective compound in the MS-based screening and least destabilizing compound in DSF. Both the overall efficacy and potency of **I** were dependent on the nucleophilicity of the C203 residue (Figures 5G and S8). At pH 7.0, a maximum efficacy of 40% inhibition was achieved, whereas at pH 8.5 a maximum efficacy of 91% inhibition was reached. Similarly, the potency of **I** increased from 6.6 equivalents to reach 50% of the observed maximum efficacy at pH 7.0, to 1.5 equivalents at pH 8.5. These results further established the covalent mode of action of **I**. The kinetics of **I** were investigated through time-dependent MS analysis (Figure 5H), revealing relatively slow binding of **I**, requiring around 2 h for 50% labeling and overnight incubation for full labeling of C203 at pH 8.0. It is hypothesized that the relatively slow covalent engagement of compound **I** results from the low warhead reactivity of the electron-deficient haloarene warhead^57^ combined with the required conformational change in NR2F6 to accommodate binding of **I**. No recruitment of canonical coregulators to the NR2F6 LBD was induced by binding of compound **I** (Figure S6B).

## Conclusions

In this work, the in vitro coregulator binding profile of the NR2F6 LBD has been characterized, confirming the direct and constitutive recruitment of F/YSXXLXXL/Y motifs derived from the BCL11A and NSD1 coregulator proteins. By combining the NR2F6-stabilizing effects of these coregulator peptides with an MBP fusion strategy, the first crystal structure of the NR2F6 nuclear receptor was elucidated. The NR2F6/NSD1 cocrystal structure revealed an autorepressed LBD conformation in which H12 folds over the collapsed LBD, generating a surface pocket that is targeted by the α-helical NSD1 peptide. Building on this structural knowledge, electron-deficient haloarene probes were identified to covalently modulate NR2F6 coregulator recruitment through preferential targeting of the C203 residue of NR2F6 located near the peptide binding interface. The data presented herein provide structural and biochemical insights into the molecular mechanisms underlying NR2F6 function that may inform future studies investigating NR2F6 biology. As there is currently a lack of chemical matter to modulate NR2F6 activity, the compounds described in this work can feature as tools to further study NR2F6 and can serve as starting points for the development of more potent, selective (covalent) NR2F6 modulators.

## Supplementary Material





## Notes

Additional figures, synthetic procedures, compound characterization, detailed description of methods concerning protein expression, in silico model generation and biochemical assays (PDF)
Compound characterization Checklist (XLS)

## Abbreviations

NR2F6: Nuclear Receptor subfamily 2 group F member 6
EAR-2: V-erbA-related protein 2
COUP-TF: chicken ovalbumin upstream promoter-transcription factor
NSD1: nuclear receptor binding SET domain protein 1
BCL11A: B-cell lymphoma/leukemia 11A
FOG2: Friend of GATA protein 2
dATRO: *Drosophila* homologue of Atrophin
NR: nuclear receptor
LBD: ligand binding domain
MBP: maltose-binding protein
H12: helix 12
TR-FRET: time-resolved FRET
DSF: differential scanning fluorimetry
Q-ToF MS: quadrupole time-of-flight mass spectrometry
SEC: size-exclusion chromatography
SER: surface entropy reduction.
