# Supplementary material for: Structural Elucidation and Covalent Modulation of the Autorepressed Orphan Nuclear Receptor NR2F6
Source: ACS Chem Biol. 2025 Sep 10;20(9):2308–17. doi: 10.1021/acschembio.5c00475 (PMC12455566; doi:10.1021/acschembio.5c00475)
Supplement: Supplementary file 1 [file cb5c00475_si_001.pdf]

Supporting Information

Structural Elucidation and Covalent Modulation of the Autorepressed Orphan Nuclear Receptor NR2F6

Guido J.M. Oerlemans[†], Maxime C.M. van den Oetelaar[†], Siebe P. van den Elzen[†], Luc Brunsveld[†,*]

AUTHOR ADDRESS

[†]Laboratory of Chemical Biology, Department of Biomedical Engineering and Institute of Complex Molecular Systems, Technische Universiteit Eindhoven, 5612 AZ Eindhoven, The Netherlands.

CONTENTS

1. Materials and Methods S4

1.1 *In silico* NR2F6 Model S4
1.2 General Chemistry S4
1.3 Synthetic Procedures S5
1.4 Protein Expression and Purification S8
1.5 X-ray Crystallography, Data Collection and Refinement S11
1.6 Biochemical Assays S11

2. Supplementary Figures and Tables

Figure S1: TR-FRET NSD1 displacement curves S15
Figure S2: Design and characterization of the MBP-NR2F6 fusion construct S16
Figure S3: Additional NR2F6 crystal structure details S17
Figure S4: Analytical SEC chromatogram of MBP-NR2F6 and BSA S19
Figure S5: $NR2F6^{E204A}$ and $NR2F6^{F211A}$ DSF and TR-FRET characterization S19
Figure S6: Profiling of proposed NR2F6 LBD binders S19
Figure S7: NR2F6 cysteine mutants and covalent probe screening S20
Figure S8: pH dependency of compound I S20
Figure S9: Protein Q-ToF MS and SDS-Page S21
Table S1: Peptide sequences S22
Table S2: Data collection and refinement statistics S23
Table S3: Covalent library screening results S24

3. NMR spectra (^{1}H & ^{13}C) and LC-UV traces for assayed compounds S26

4. Supporting References S36

SUPPORTING INFORMATION

1. Materials and Methods

1.1 *In silico* NR2F6 model

To model the hypothetical active conformation of the NR2F6 LBD, a homology model was constructed based on a high-resolution agonist-bound RXRα LBD structure (PDB: 6LB4) using the SWISS-MODEL homology-modelling server[1–10]. The open-source program fpocket[11] was used to predict the ligand binding pocket volume.

1.2 General Chemistry

All solvents were supplied by Biosolve and used without further purification. Dry solvent was obtained from a MBRAUN Solvent Purification System (MB-SPS-800). Water was purified by a Millipore purification train. Deuterated solvents were obtained from Cambridge isotope Laboratories. Solvents were removed under reduced pressure using a Büchi rotary evaporator and a diaphragm pump. All reagents were commercially available and were supplied by Sigma-Aldrich and BLD Pharm. Proton (^{1}H) NMR (400 MHz), carbon (^{13}C) NMR (100 MHz), fluor (^{19}F) NMR (400 MHz) and 2D NMR (400 MHz) were recorded on a Bruker Avance 400 MHz spectrometer. Proton spectra are referenced to tetramethyl silane (TMS). Carbon spectra are referenced to TMS or the solvent peak of the deuterated spectrum. NMR spectra are reported as follows: chemical shift (δ) in parts per million (ppm), multiplicity (s = singlet, d = doublet, t = triplet, q = quartet, m = multiplet, dd = doublet of doublet, td = triplet of doublets), coupling constant (J) in Hertz (Hz) (if applicable) and integration (proton spectra only). Peak assignments are based on additional 2D NMR techniques (COSY, HMBC, HSQC). Analytical Liquid Chromatography coupled with Mass Spectrometry (LC-MS) was performed on a C4 Jupiter SuC4300A 150 x 2.0 mm column using ultrapure water with 0.1% formic acid (FA) and acetonitrile with 0.1% FA, in general with a gradient of 5% to 100% acetonitrile over 10 minutes, connected to a Thermo Fischer LCQ Fleet Ion Trap Mass Spectrometer. The purity of the samples was assessed using a UV detector at 254 nm. Unless stated otherwise all final compounds were >95% pure as judged by HPLC. High resolution mass spectra (HRMS) were recorded using a Waters ACQUITY UPLC I-Class LC system coupled to a Xevo G2 Quadrupole Time of Flight (Q-TOF) mass spectrometer. Flash column chromatography was performed on a Biotage Isolera One, using pre-packed FlashPure Büchi silica columns with 40 μm particle size and the sample was dry loaded on Celite®. Reaction progress was monitored by thin-layer liquid chromatography (TLC) using Merck TLC silica gel 60 F254 plates. Visualization of the plates was achieved using an ultraviolet lamp (λmax = 254 nm). Peptides were ordered from Genscript with ≥ 95% purity. 9-cis-retinoic acid was ordered from Sigma Aldrich. Troglitazone was ordered from MedChemExpress.

1.3 Synthetic Procedures

The synthesis of a part of the library has been described elsewhere.[12]

1.3.1 General Procedure for Amide Coupling

2-chloro-5-nitrobenzoyl chloride (500 mg, 2.27 mmol) or 4-chloro-3-nitrobenzoyl chloride (500 mg, 2.27 mmol) was dissolved in dry pyridine (2.5 mL) and cooled to 0° C under argon. The aniline derivative (2.95 mmol) was added, and the resulting reaction mixture was stirred overnight at room temperature. Subsequently, the reaction mixture was poured on ice, and the precipitate was filtered and washed with cold water. After drying, the resulting solid was recrystallized from boiling ethanol or ethyl acetate/hexane to obtain the title compound.

1.3.2 4-chloro-3-nitro-*N*-phenylbenzamide

According to the General Procedure for Amide Coupling, aniline was reacted with 4-chloro-3-nitrobenzoyl chloride to afford the title compound (389 mg, 62%). ^{1}H NMR (400 MHz, Acetone-d_6) δ 9.87 (s, 1H), 8.56 (d, J = 2.2 Hz, 1H), 8.27 (dd, J = 8.4, 2.2 Hz, 1H), 7.83 (d, J = 8.4 Hz, 1H), 7.80 (d, J = 7.9 Hz, 1H), 7.36 (t, J = 7.9 Hz, 2H), 7.14 (t, J = 7.5 Hz, 1H); ^{13}C NMR (100 MHz, Acetone-d_6) δ 163.26, 148.74, 139.58, 136.22, 133.18, 132.85, 129.56, 125.43, 125.17, 121.21, 121.12. LC-MS (ESI): calc. for $C_{13}H_{10}ClN_2O_3$ $[M+H]^+$: 277.03, observed: 277.50 (R_t = 4.30 min). HRMS (ESI): calc. for $C_{13}H_{10}ClN_2O_3$ $[M+H]^+$: 277.0380, observed: 277.0380.

1.3.3 2-chloro-*N*-(4-fluorophenyl)-5-nitrobenzamide

According to the General Procedure for Amide Coupling, 4-fluoroaniline was reacted with 2-chloro-5-nitrobenzoyl chloride to afford the title compound (401 mg, 60%). ^{1}H NMR (400 MHz, Acetone-d_6) δ 9.86 (s, 1H), 8.50 (d, J = 2.7 Hz, 1H), 8.33 (dd, J = 8.8, 2.7 Hz, 1H), 7.86 – 7.76 (m, 3H), 7.17 (t, J = 8.8 Hz, 2H); ^{13}C NMR (100 MHz, Acetone-d_6): δ. 163.64, 160.22 (d, J = 242.0 Hz), 147.46, 138.68, 138.46, 135.80 (d, J = 3.0 Hz), 132.26, 126.50, 124.84, 122.67 (d, J = 8.0 Hz), 116.25 (d, 22.5 Hz). LC-MS (ESI): calc. for $C_{13}H_9ClFN_2O_3$ $[M+H]^+$: 295.02, observed: 295.67 (R_t = 4.14 min). HRMS (ESI): calc. for $C_{13}H_9ClFN_2O_3$ $[M+H]^+$: 295.0286, observed: 295.0286.

1.3.4 4-chloro-*N*-(2-fluorophenyl)-3-nitrobenzamide

According to the General Procedure for Amide Coupling, 2-fluoroaniline was reacted with 4-chloro-3-nitrobenzoyl chloride to afford the title compound (473 mg, 71%). ^{1}H NMR (400 MHz, Acetone-d_6) δ 9.63 (s, 1H), 8.60 (d, J = 2.1 Hz, 1H), 8.30 (dd, J = 8.4, 2.1 Hz, 1H), 8.08 – 7.96 (m, 1H), 7.89 (d, J = 8.4 Hz, 1H), 7.31 – 7.17 (m, 3H); ^{13}C NMR (100 MHz, Acetone-d_6) δ 163.62, 155.64 (d, J = 246.1 Hz), 148.84,

135.63, 133.34, 132.95, 129.86, 127.25 (d, *J* = 7.7 Hz), 126.71 (d, *J* = 11.7 Hz), 125.94 (d, *J* = 1.4 Hz), 125.72, 125.19 (d, *J* = 3.7 Hz), 116.32 (d, *J* = 19.8 Hz). LC-MS (ESI): calc. for $C_{13}H_9ClFN_2O_3$ [M+H]$^+$: 295.02, observed: 295.58 (R_t = 4.24 min). HRMS (ESI): calc. for $C_{13}H_9ClFN_2O_3$ [M+H]$^+$: 295.0286, observed: 295.0288.

1.3.5 4-chloro-*N*-(4-fluorophenyl)-3-nitrobenzamide

According to the General Procedure for Amide Coupling, 4-fluoroaniline was reacted with 4-chloro-3-nitrobenzoyl chloride to afford the title compound (460 mg, 69%). ^{1}H NMR (400 MHz, Acetone-*d*$_6$) δ 9.92 (s, 1H), 8.56 (d, *J* = 2.2 Hz, 1H), 8.28 (dd, *J* = 8.4, 2.2 Hz, 1H), 7.87 (d, *J* = 8.4 Hz, 1H), 7.85 – 7.80 (m, 2H), 7.19 – 7.09 (m, 2H); ^{13}C NMR (100 MHz, Acetone-*d*$_6$) δ 163.23, 160.18 (d, *J* = 241.4 Hz), 148.82, 136.10, 135.90 (d, *J* = 2.9 Hz), 133.20, 132.94, 129.68, 125.44, 123.14 (d, *J* = 7.7 Hz), 116.10 (d, *J* = 22.4 Hz). LC-MS (ESI): calc. for $C_{13}H_9ClFN_2O_3$ [M+H]$^+$: 295.02, observed: 295.67 (R_t = 4.35 min). HRMS (ESI): calc. for $C_{13}H_9ClFN_2O_3$ [M+H]$^+$: 295.0286, observed: 295.0290.

1.3.6 2-chloro-5-nitro-*N*-(4-(trifluoromethyl)phenyl)benzamide

According to the General Procedure for Amide Coupling, 4-(trifluoromethyl)aniline was reacted with 2-chloro-5-nitrobenzoyl chloride to afford the title compound (454 mg, 58%). ^{1}H NMR (400 MHz, Acetone-*d*$_6$) δ 10.14 (s, 1H), 8.55 (d, *J* = 2.7 Hz, 1H), 8.36 (dd, *J* = 8.8, 2.7 Hz, 1H), 8.01 (d, *J* = 8.9 Hz, 2H), 7.85 (d, *J* = 8.8 Hz, 1H), 7.75 (d, *J* = 8.9 Hz, 2H); ^{13}C NMR (100 MHz, Acetone-*d*$_6$) δ 164.14, 147.50, 142.94, 138.48, 138.34, 132.36, 127.05 (q, *J* = 3.8 Hz), 126.76, 126.37 (q, *J* = 32.4 Hz), 125.33 (q, *J* = 270.7 Hz), 124.94, 120.73. LC-MS (ESI): calc. for $C_{14}H_7ClF_3N_2O_3$ [M-H]$^-$: 343.02, observed: 343.42 (R_t = 4.58 min). HRMS (ESI): calc. for $C_{14}H_7ClF_3N_2O_3$ [M+H]$^+$: 345.0254, observed: 345.0258.

1.3.7 2-chloro-*N*-(4-ethoxyphenyl)-5-nitrobenzamide

According to the General Procedure for Amide Coupling, 4-ethoxyanline was reacted with 2-chloro-5-nitrobenzoyl chloride to afford the title compound (437 mg, 60%). ^{1}H NMR (400 MHz, Chloroform-*d*) δ 8.57 (d, *J* = 2.7 Hz, 1H), 8.23 (dd, *J* = 8.8, 2.7 Hz, 1H), 7.83 (s, 1H), 7.63 (d, *J* = 8.8 Hz, 1H), 7.51 (d, *J* = 9.0 Hz, 2H), 6.91 (d, *J* = 9.0 Hz, 2H), 4.05 (q, *J* = 7.0 Hz, 2H), 1.43 (t, *J* = 7.0 Hz, 3H); ^{13}C NMR (100 MHz, $CDCl_3$) δ 162.04, 156.66, 146.63, 137.56, 136.67, 131.55, 129.76, 125.84, 125.31, 122.20, 114.97, 63.78, 14.82. LC-MS (ESI): calc. for $C_{15}H_{14}ClN_2O_4$ [M+H]$^+$: 321.06, observed: 321.58 (R_t = 4.24 min). HRMS (ESI): calc. for $C_{15}H_{14}ClN_2O_4$ [M+H]$^+$: 321.0642, observed: 321.0643.

1.3.8 2-chloro-*N*-(3-hydroxyphenyl)-5-nitrobenzamide

3-aminophenol (744 mg, 6.82 mmol) was dissolved in THF (3 mL) and cooled to 0˚ C under argon. 2-chloro-5-nitrobenzoyl chloride (500 mg, 2.27 mmol) was dissolved in dry THF (2 mL) and added dropwise to this solution. The reaction mixture was stirred overnight at room temperature, quenched with 5% aqueous hydrochloric acid (2 mL), poured into ice and washed with 5% aqueous hydrochloric acid (10 mL) followed by water (10 mL). After drying, the solid was recrystallized from boiling ethyl acetate to afford the title compound (150 mg, 23%). ^{1}H NMR (400 MHz, Acetone-d_6) δ 8.47 (d, J = 2.8 Hz, 1H), 8.31 (dd, J = 8.8, 2.8 Hz, 1H), 7.80 (d, J = 8.8 Hz, 1H), 7.50 – 7.44 (m, 1H), 7.20 – 7.14 (m, 2H), 6.71 – 6.61 (m, 1H); ^{13}C NMR (100 MHz, Acetone-d_6) δ 163.55, 158.61, 147.39, 140.47, 138.82, 138.41, 132.18, 130.51, 126.37, 124.78, 112.29, 111.76, 107.76. LC-MS (ESI): calc. for $C_{13}H_{10}ClN_2O_4$ [M+H]$^+$: 293.03, observed: 293.50 (R_t = 3.54 min). HRMS (ESI): calc. for $C_{13}H_{10}ClN_2O_4$ [M+H]$^+$: 293.0329, observed: 293.0322.

1.3.9 2-chloro-*N*-(4-hydroxyphenyl)-5-nitrobenzamide

4-aminophenol (744 mg, 6.82 mmol) was dissolved in THF (3 mL) and cooled to 0˚ C under argon. 2-chloro-5-nitrobenzoyl chloride (500 mg, 2.27 mmol) was dissolved in dry THF (2 mL) and added dropwise to this solution. The reaction mixture was stirred for three hours at 0˚C, quenched with 5% aqueous hydrochloric acid (2.3 mL), poured into ice and washed with 5% aqueous hydrochloric acid (10 mL) followed by water (10 mL). After drying, the solid was recrystallized from boiling ethyl acetate to afford the title compound (312 mg, 47%). ^{1}H NMR (400 MHz, Acetone-d_6) δ 9.59 (s, 1H), 8.46 (d, J = 2.8 Hz, 1H), 8.31 (dd, J = 8.8, 2.8 Hz, 1H), 7.81 (d, J = 8.8 Hz, 1H), 7.60 (d, J = 8.9 Hz, 2H), 6.85 (d, J = 8.9 Hz, 2H); ^{13}C NMR (100 MHz, Acetone-d_6) δ 163.22, 155.24, 147.46, 139.14, 138.48, 132.19, 131.64, 126.26, 124.80, 122.53, 116.15. LC-MS (ESI): calc. for $C_{13}H_{10}ClN_2O_4$ [M+H]$^+$: 293.03, observed: 293.58 (R_t = 3.38 min). HRMS (ESI): calc. for $C_{13}H_{10}ClN_2O_4$ [M+H]$^+$: 293.0329, observed: 293.0328.

1.3.10 *Tert*-butyl (4-(2-chloro-5-nitrobenzamido)phenyl)carbamate

2-chloro-5-nitrobenzoyl chloride (500 mg, 2.27 mmol) was dissolved in dry pyridine (2.5 mL) and cooled to 0˚ C under argon. *Tert*-butyl (4-aminophenyl)carbamate (616 mg, 2.95 mmol) was added, and the resulting reaction mixture was stirred overnight at room temperature. The reaction mixture was poured on ice and the precipitate was filtered and washed with cold water. The crude precipitate was purified via flash column chromatography (0-60% EtOAc in n-heptane) to afford the title compound (513 mg, 58%). 1H NMR (400 MHz, DMSO-d6) δ 10.58 (s, 1H), 9.33 (s, 1H), 8.44 (d, J = 2.8 Hz, 1H), 8.33 (dd, J = 8.9, 2.8 Hz, 1H), 7.88 (d, J = 8.9 Hz, 1H), 7.58 (d, J = 9.0 Hz, 2H), 7.44 (d, J = 9.0 Hz, 2H), 1.48 (s, 9H). 13C NMR (100 MHz, DMSO-d6) δ 162.27, 152.70, 146.03, 137.74, 137.03, 135.79, 132.81, 131.22, 125.49, 123.76,

120.19, 118.33, 78.86, 28.06. LC-MS (ESI): calc. for $C_{18}H_{17}ClN_3O_5$ $[M-H]^-$: 390.09, observed: 390.50 (Rt = 4.39 min).

1.3.11 *N*-(4-aminophenyl)-2-chloro-5-nitrobenzamide (TFA salt)

Tert-butyl (4-(2-chloro-5-nitrobenzamido)phenyl)carbamate (200 mg, 0.51 mmol) was dissolved in a mixture of DCM:TFA (70:30, 2 mL) and stirred for 5 hours at room temperature. The mixture was concentrated under reduced pressure, and the solid was recrystallized from boiling ethyl acetate to afford the title compound (128 mg, 85%). 1H NMR (400 MHz, Methanol-d4) δ 8.39 (d, J = 2.7 Hz, 1H), 8.30 (dd, J = 8.8, 2.7 Hz, 1H), 7.76 (d, J = 8.8 Hz, 1H), 7.43 (d, J = 8.8 Hz, 2H), 6.79 (d, J = 8.8 Hz, 2H); 13C NMR (101 MHz, Methanol-d4) δ 165.27, 147.93, 145.06, 139.15, 138.98, 132.46, 130.74, 126.60, 124.89, 123.20, 117.20. LC-MS (ESI): calc. for $C_{13}H_{11}ClN_3O_3$ $[M+H]^+$: 292.04, observed: 292.58 (Rt = 1.54 min). HRMS (ESI): calc. for $C_{13}H_{11}ClN_3O_3$ $[M+H]^+$: 292.0489, observed: 292.0475.

1.3.12 6-chloro-3-nitro-*N*-phenylpyridin-2-amine

2,6-dichloro-3-nitropyridine (100 mg, 0.52 mmol) was dissolved in dry DCM (5 mL) and cooled to 0˚ C under argon, followed by the addition of triethylamine (157 mg, 220 µL, 1.55 mmol). Subsequently, aniline (48 mg, 50 µL, 0.52 mmol) was added dropwise. The reaction mixture was stirred overnight at room temperature, diluted with water (10 mL) and extracted with DCM (3x 10 mL). The combined organic layer was washed with water (2x 20 mL) and brine, dried over anhydrous sodium sulfate, filtered and concentrated under reduced pressure. The crude material was purified via flash column chromatography (0-20% EtOAc in n-heptane) to afford the title compound as a red solid (68 mg, 53%). 1H NMR (400 MHz, Chloroform-d) δ 10.23 (s, 1H), 8.42 (d, J = 8.7 Hz, 1H), 7.63 (d, J = 7.4 Hz, 2H), 7.44 – 7.32 (m, 2H), 7.18 (t, J = 7.5 Hz, 1H), 6.76 (d, J = 8.7 Hz, 1H); 13C NMR (100 MHz, Chloroform-d) δ 155.25, 148.23, 136.82, 136.19, 128.03, 126.11, 124.09, 120.94, 113.01. LC-MS (ESI): calc. for $C_{11}H_9ClN_3O_2$ $[M+H]^+$: 250.03, observed: 250.17 (Rt = 4.64 min). HRMS (ESI): calc. for $C_{11}H_9ClN_3O_2$ $[M+H]^+$: 250.0383, observed: 250.0381.

1.4 Protein Expression and Purification

1.4.1 Expression of His_6-SUMO-NR2F6 LBD and mutants

A pET-28a(+) expression vector encoding the human NR2F6 LBD (residues 164-404) with an N-terminal His_6-SUMO-tag derived from yeast SMT3 was transformed by heat shock into BL21(DE3) *E.coli* cells. A single colony was used to inoculate 40 mL of autoclaved LB-medium containing 50 µg/mL kanamycin. After overnight incubation at 37 ˚C 250 rpm, the pre-culture was transferred to 2L of Terrific Broth medium supplemented with kanamycin (50 µg/mL) and 0.5 mM $MgCl_2$. The culture was incubated at 37 ˚C 150

rpm until an OD_{600} of 0.8-1.0 was reached. The culture was subsequently cooled for 2 hours at 4 ˚C. Protein expression was then induced through addition of 20 µM isopropyl β-d-1-thiogalactopyranoside (IPTG), and cultures were incubated for 16 hours at 26 ˚C 150 rpm. Cells were collected through centrifugation (10,000 RCF, 10 minutes) and resuspended in lysis buffer (50 mM Tris pH 8.0, 500 mM NaCl, 20 mM imidazole, 5 mM 2-mercaptoethanol). The lysate was supplemented with cOmplete™, EDTA-free Protease Inhibitor Cocktail tablets (1 tablet/100 mL) and Benzonase® nuclease (0.1 µL/mL). The cells were lysed using a homogenizer (Avestin Emulsiflex C3) at 20,000 psi and the lysate was cleared by centrifugation (40,000 RCF, 30 minutes). The supernatant was filtered over a 0.2 µM PES filter (Supor®) and loaded onto a pre-equilibrated His-trap HP column (10 mL resin, Cytiva). The column was washed with lysis buffer (10 column volumes), wash buffer (50 mM Tris pH 8.0, 500 mM NaCl, 50 mM imidazole, 5 mM 2-mercaptoethanol; 10 column volumes), and the protein was eluted with elution buffer (50 mM Tris pH 8.0, 500 mM NaCl, 200 mM imidazole, 5 mM 2-mercaptoethanol). Fractions containing the protein of interest were combined and dialyzed against storage buffer (20 mM Tris pH 8.0, 150 mM NaCl, 1 mM DTT), concentrated to 20 mg/mL, flash frozen and stored at -80 ˚C.

1.4.2 Expression of His_6-NR2F6 LBD

A pET-15b expression vector encoding the human NR2F6 LBD (residues 164-404) with an N-terminal His_6-tag was transformed by heat shock into BL21(DE3) *E.coli* cells. A single colony was used to inoculate 40 mL of LB-media containing 100 µg/mL ampicillin. After overnight incubation at 37 ˚C 250 rpm, the pre-culture was transferred to 2L of Terrific Broth supplemented with ampicillin (100 µg/mL) and 0.5 mM $MgCl_2$. The culture was incubated at 37 ˚C 150 rpm until an OD_{600} of 0.8-1.0 was reached. Protein expression was induced through addition of 500 µM isopropyl β-d-1-thiogalactopyranoside (IPTG), and cultures were incubated for 16 hours at 18 ˚C 150 rpm. Cells were collected through centrifugation (10,000 RCF, 10 minutes) and resuspended in lysis buffer (50 mM Tris pH 7.3, 300 mM NaCl, 10 v/v% glycerol, 20 mM imidazole, 5 mM 2-mercaptoethanol).The lysate was supplemented with cOmplete™, EDTA-free Protease Inhibitor Cocktail tablets (1 tablet/50 mL) and Benzonase® nuclease (0.1 µL/mL). The cells were lysed using a homogenizer (Avestin Emulsiflex C3) at 20,000 psi and the lysate was cleared by centrifugation (40,000 RCF, 30 minutes) and loaded onto a pre-equilibrated Ni-NTA Superflow cartridge (5 mL, Qiagen). The column was washed with lysis buffer (10 column volumes), wash buffer (50 mM Tris pH 7.3, 300 mM NaCl, 10 v/v% glycerol, 40 mM imidazole, 5 mM 2-mercaptoethanol; 10 column volumes), and the protein was eluted with elution buffer (50 mM Tris pH 7.3, 300 mM NaCl, 10 v/v% glycerol, 200 mM imidazole, 5 mM 2-mercaptoethanol). Fractions containing the protein of interest were combined and dialyzed against storage buffer (20 mM Tris pH 7.3, 150 mM NaCl, 10 v/v% glycerol, 2 mM DTT), concentrated to 2 mg/mL, flash frozen and stored at -80 ˚C.

1.4.3 Expression of MBP-NR2F6 LBD

A pET-28a(+) expression vector encoding the His_6-TEV-MBP-NR2F6 fusion protein (NR2F6 residues 199-393) was transformed by heat shock into BL21(DE3) *E.coli* cells. A single colony was used to inoculate 40 mL of LB-media containing 50 µg/mL kanamycin. After overnight incubation at 37 ˚C 250 rpm, the pre-culture was transferred to 2L of Terrific Broth supplemented with kanamycin (50 µg/mL) and 0.5 mM $MgCl_2$. The culture was incubated at 37 ˚C 150 rpm until an OD_{600} of 0.8-1.0 was reached. Protein expression was induced through addition of 30 µM isopropyl β-d-1-thiogalactopyranoside (IPTG), and cultures were incubated for 16 hours at 18 ˚C 150 rpm. Cells were collected through centrifugation (10,000 RCF, 10 minutes) and resuspended in lysis buffer (50 mM Tris pH 8.0, 500 mM NaCl, 20 mM imidazole, 2 mM 2-mercaptoethanol). The lysate was supplemented with cOmplete™, EDTA-free Protease Inhibitor Cocktail tablets (1 tablet/100 mL) and Benzonase® nuclease (0.1 µL/mL). The cells were lysed using a homogenizer (Avestin Emulsiflex C3) at 20,000 psi and the lysate was cleared by centrifugation (40,000 RCF, 30 minutes) and loaded onto a His-trap HP column (10 mL resin, Cytiva). The column was washed with lysis buffer (10 column volumes), wash buffer (50 mM Tris pH 8.0, 500 mM NaCl, 40 mM imidazole, 2 mM 2-mercaptoethanol; 10 column volumes), and the protein was eluted with elution buffer (50 mM Tris pH 8.0, 500 mM NaCl, 200 mM imidazole, 2 mM 2-mercaptoethanol). Fractions containing the protein of interest were combined and dialyzed against 20 mM Tris 8.0, 150 mM NaCl, 2 mM BME for four hours. Subsequently, TEV protease was added (1 mg TEV/10 mg of purified protein) to remove the purification tag, and the protein was dialyzed against 20 mM Tris pH 8.0, 100 mM NaCl, 1 mM TCEP overnight. The cleaved sample was loaded onto a pre-equilibrated His-trap HP column (5 mL resin, Cytiva) and the flowthrough was purified via size exclusion chromatography (HiLoad 16/600 Superdex 200pg) with crystallography buffer (10 mM Hepes pH 7.0, 50 mM NaCl, 5 mM maltose, 1 mM TCEP) as running buffer. Fractions containing the protein of interest were concentrated to 12-15 mg/mL, flash frozen and stored at -80 ˚C.

1.4.4 Analytical SEC of MBP-NR2F6 LBD

Analytical SEC was performed on a BioRad NGC system at 4 °C. Protein samples (1 mg/mL) were injected onto a Superdex 200 Increase 10/300 GL column (Cytiva) pre-equilibrated with SEC buffer (10 mM Hepes pH 7.0, 50 mM NaCl, 5 mM maltose, 0.5 mM TCEP). Proteins were eluted at a flow rate of 0.2 mL/min and monitored by UV absorbance at 280 nm.

1.4.5 Site-directed Mutagenesis

Point mutations (F211A, E204A, C203A, C304L/C316S) were introduced using the QuikChange Lightning Multi Site-Directed Mutagenesis Kit (Agilent) in accordance with supplier recommended protocols.

Primers were purchased from Integrated DNA Technologies. Mutant DNA was isolated using the QIAprep® Spin Miniprep Kit (Qiagen) in accordance with the supplied protocol. Mutations were confirmed by sequencing (Baseclear).

1.4.6 Q-ToF MS

The purity and exact mass of the expressed proteins were determined using a High-Resolution LC-MS system consisting of a Waters ACQUITY UPLC I-Class system coupled to a Xevo G2 Quadrupole Time-of-Flight (Q-ToF) mass spectrometer operated in positive electrospray ionization (ESI) mode, scanning between 500-2000 m/z. The protein was separated with a Polaris 3 C18-A reverse phase column (2.0 x 100 mm, Agilent) using a flowrate of 0.3 mL/min and a gradient of 15-75% acetonitrile in water, supplemented with 0.1% (v/v) formic acid. The MaxENT1 algorithm in the Masslynx v4.1 (SCN862) software was used to deconvolute the m/z spectra. Q-ToF MS spectra of the proteins used in this study are listed in Figure S9.

1.5 X-ray Crystallography, Data Collection and Refinement

Ac-NSD1 was dissolved in crystallography buffer to a final concentration of 5 mM. MBP-NR2F6 (10 mg/mL) was incubated with 2.5 equivalents of ac-NSD1 peptide in crystallography buffer for 1 hour on ice. The sample was centrifuged at 20,000 RCF for 20 minutes at 4˚C to remove precipitate. Crystals of the NR2F6/NSD1 complex were grown at 20 ˚C in sitting drops containing 250 nL complex solution and 250 nL 90 mM SPG pH 8.0, 22.5% w/v PEG1500, 100 mM potassium chloride. Crystals grew within three days and were cryoprotected after 14 days using mother liquors supplemented with 25% (v:v) glycerol and flash frozen in liquid nitrogen. X-ray diffraction data was collected at the ESRF beamline ID23-1 (Grenoble, France). AutoPROC[13] was used to integrate the data and AIMLESS[14,15] (CCP4i2 suite[16]) was used for scaling. The data was phased using PHASER[17], using AlphaFold2 Colab[18,19] generated structures of MBP and NR2F6 LBD as individual search models for molecular replacement, and ligand restraints were generated using AceDRG[20]. REFMAC[21], COOT[22] and PDB-REDO[23] were used for subsequent refinement and model building. Final refinement was performed using phenix.refine[24] from the Phenix software suite[25]. Pymol (version 2.2.3, Schrödinger) was used to make the figures, and the structure was deposited in the protein data bank (PDB) with ID: 8C5L. See Table S2 for data collection and refinement statistics.

1.6 Biochemical Assays

1.6.1 NSD1 Displacement by Acetylated Peptides (TR-FRET)

250 nM N-terminal biotinylated NSD1 coregulator peptide and 20 nM His_6-NR2F6-LBD were incubated in the presence of 0.66 nM terbium-labeled anti-His antibody (Revvity, 61HISTLB) and 31.25 nM D2-labeled streptavidin (Revvity, 610SADLB) in TR-FRET assay buffer (10 mM Hepes, 150 mM NaCl, 5 mM

DTT, 0.1 mM CHAPS and 0.1% BSA (w/v), pH 7.5). Acetylated peptides (10 mM in DMSO) were titrated using a 2-fold dilution series in white, low volume, non-binding 384-well plates (Corning, 4513) at a final volume of 10 µL, keeping the final DMSO concentration at 1% throughout. Plates were incubated for one hour at room temperature and measured on a Tecan Spark plate reader (λ_{ex}: 340 nm, λ_{em}: 620 nm and 665 nm) using the supplier recommended HTRF settings. Data was analyzed using GraphPad Prism 10 and fitted using a four-parameter logistic equation. Data were recorded in triplicate; data shown is representative of three independent experiments, average and standard deviation of three independent experiments.

1.6.2 NR2F6 Coregulator Profiling (TR-FRET)

Assay was performed in analogous fashion to the TR-FRET protocol described previously, using various N-terminal biotinylated coregulator peptides (250 nM; sequences listed in Table S1) and 20 nM His_6-NR2F6-LBD or (mutant) His_6-SUMO-NR2F6 LBD. For compound studies, 20 nM (ligated) His_6-SUMO-NR2F6 LBD was incubated with DMSO or a fixed concentration of 50 µM non-covalent ligand was used. Specific signal (ΔR) is the 665nm/620nm ratio of protein with peptide minus the 665nm/620nm ratio of the peptide background. Data recorded in triplicate.

1.6.3 Bio-NSD1 Titration (TR-FRET)

Assay was performed in analogous fashion to the TR-FRET protocol described previously. N-terminal biotinylated NSD1 peptide/D2-labeled streptavidin (fixed 8:1 ratio) was titrated to 20 nM His_6-NR2F6-LBD and 0.66 nM terbium-labeled anti-His antibody in TR-FRET assay buffer. Specific signal (ΔR) is the 665nm/620nm ratio for biotinylated NSD1 peptide/D2-labeled streptavidin minus the 665nm/620nm ratio of the D2-labeled streptavidin background. Data shown is the average and standard deviation of three independent experiments.

1.6.4 NSD1 Displacement by Covalent Probes (TR-FRET)

Assay was performed in analogous fashion to the TR-FRET protocol described previously, using 25 nM of pre-incubated His_6-SUMO-NR2F6 LBD. Data shown is the average and standard deviation of three independent experiments.

1.6.5 Differential Scanning Fluorimetry (DSF)

Differential Scanning Fluorimetry (DSF) assays were performed using 40 µL samples containing 2.5 µM (ligated) His_6-SUMO-NR2F6 LBD or MBP-NR2F6 and 10x ProteOrange (Lumiprobe, 5000X stock in DMSO) in DSF buffer (25 mM Hepes, 150 mM NaCl, 100, 2 mM DTT, pH 7.5). Acetylated peptides (10 mM in DMSO) were used at a final concentration of 100 µM, 1% DMSO. Samples were heated from 25

˚C to 80 ˚C at a rate of 0.3 ˚C per 15 s in a CFX96 Touch Real-Time PCR Detection System (Bio-Rad). Fluorescence intensity was determined using the 470/40 nm excitation and 570/20 nm emission filters. Based on these melting curves, the negative derivative melting curve was obtained from which the melting temperature was determined. Data was analyzed using GraphPad Prism 10 and reported as the change in melting temperature (ΔT_m in °C) relative to the control. ΔT_m is positive unless stated otherwise. Data shown is the average and standard deviation of three (for peptide stabilization) or two (for NR2F6 mutant ΔT_m) independent experiments.

1.6.6 Fluorescence Anisotropy (FA)

(Mutant) His_6-SUMO-NR2F6 LBD was titrated in a 2-fold dilution series starting at 283 µM to 100 nM of fluorescein amidite (FAM)-labeled NSD1 peptide in FA-buffer (25 mM Tris, 150 mM NaCl, 0.01% (v/v) Tween-20, 0.1% (w/v) BSA, pH 8.0). Dilution series were made in black, low-volume, non-binding 384-well plates (Corning 4514) at a final volume of 10 µL. Plates were incubated for one hour at room temperature, and measured on a Tecan Spark plate reader (λ_{ex}: 485 ± 20 nm, λ_{em}: 535 ± 25 nm; mirror: automatic; flashes: 30; settle time: 1 ms; gain: optimal; Z-position: calculated from well). Wells containing only 100 nM FAM-NSD1 were used to determine the G-factor. Data was analyzed using GraphPad Prism 10 and fitted using a four-parameter logistic equation. Data shown is the average and standard deviation of three independent experiments.

1.6.7 NR2F6 Ligation

Covalent probes were incubated overnight with 20 µM (mutant) His_6-SUMO-NR2F6 LBD in buffer containing 50 mM NaCl, 50 mM Tris pH 8.0 (or pH 7.0, 7.5 and 8.5 for the pH screen), 2 mM DTT, 10 mM CHAPS and 2% DMSO at the desired excess (100 µM for MS screening ,1.4-fold dilution series from 400 µM for TR-FRET dose-response assays and 400 µM for DSF experiments). For Q-ToF analysis, samples were directly diluted in MQ with 0.1% (v/v) formic acid. For TR-FRET experiments, samples were diluted in TR-FRET buffer prior to transfer to TR-FRET buffer containing detection reagents. For DSF experiments, samples were rebuffered into DSF buffer using a PD Spin Trap G-25 column (Cytiva) to remove excess compound.

1.6.8 MS-based Library Screening and time-dependent Q-ToF MS

Overnight incubated His_6-SUMO-NR2F6 LBD samples (5-fold compound excess) were diluted in MQ with 0.1% (v/v) formic acid and transferred to conical LC-MS vials, snap frozen in liquid nitrogen and stored at -80 ˚C prior to analysis using the Q-ToF MS procedure described previously. The degree of NR2F6 labeling by each compound was quantified using peak intensities of the deconvoluted protein spectra:

$Intensity_{NR2F6+compound} / (Intensity_{NR2F6+compound} + Intensity_{NR2F6\ apo})*100\%$

In the case of any double labeling of $NR2F6^{C203A}$, the intensity of the 1X and 2X bound peaks were combined as $intensity_{NR2F6+compound}$ to quantify off-target binding. Data was analyzed using GraphPad Prism 10. Data shown is of a single incubation experiment per compound. For the time-dependent Q-ToF MS, a similar approach was followed using 20-fold compound excess, sampling at t: 0h, 1h, 2h, 4h, 6h, 8h and 20h. Data was analyzed using GraphPad Prism 10. Data shown is the average and standard deviation of three independent experiments.

2. Supplementary Figures and Tables

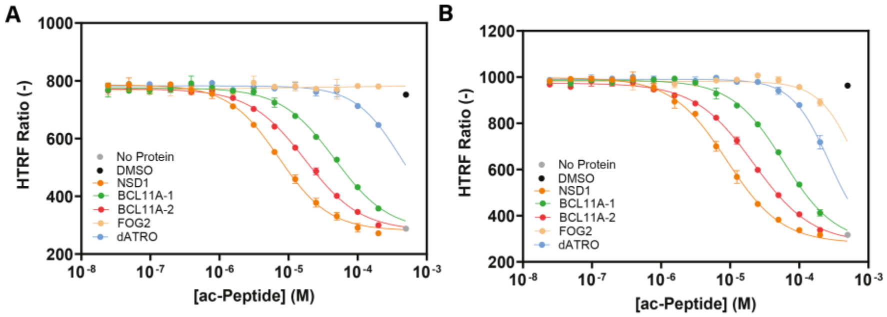

Figure S1A & B. Replicate concentration-response curves of acetylated NSD1, BCL11A-1, BCL11A-2, FOG2 and dATRO in a bio-NSD1 displacement TR-FRET assay. Data recorded in technical triplicate.

A

```
                                         A/G repeat          ▼: : * :**:** :::**:
NR2F6        ----QPVSELIAQLLRAEPYPAAAGRFGAGGGAAGAVLGIDNVCELAARLLFSTVEWARH  219
COUP-TF2     LNCHSYLSGYISLLLRAEPYPT--SRFGSQCMQPNNIMGIENICELAARMLFSAVEWARN  229
TLX          ELAAVSTTPERQTLVSLAQPTPKYPHEVNGTPMYLYEVATESVCESAARLLFMSIKWAKS  202
PNR          ITAETCAKLEPEDADENIDVTSNDPEFPSSPYSSSSPCGLDSIHETSARLLFMAVKWAKN  237
                    H1                                 H3

              * *  *   **: **  :* ***:*   * ::*:    ***              :
NR2F6        APFFPELPVADQVALLRLSWSELFVLNAAQAALPLHTAPLLAAAGLHAAPMAAERAVAFM  279
COUP-TF2     IPFFPDLQITDQVALLRLTWSELFVLNAAQCSMPLHVAPLLAAAGLHASPMSADRVVAFM  289
TLX          VPAFSTLSLQDQLMLLEDAWRELFVLGIAQWAIPVDANTLLAVSGMNGDNTDSQKLNKII  262
PNR          LPVFSSLPFRDQVILLEEAWSELFLLGAIQWSLPLDSCPLLAPPEASAAGGAQGRLTLAS  297
              H3'        H4-5

                : :** : ::  * :* :*: *:* :  *                 : :   : **:::*
NR2F6        DQVRAFQEQVDKLGRLQVDSAEYGCLKAIALFTPD-------ACGLSDPAHVESLQEKAQ  332
COUP-TF2     DHIRIFQEQVEKLKALHVDSAEYSCLKAIVLFTSD-------ACGLSDVAHVESLQEKSQ  342
TLX          SEIQALQEVVARFRQLRLDATEFACLKCIVTFKAVPTHSGSELRSFRNAAAIAALQDEAQ  322
PNR          METRVLQETISRFRALAVDPTEFACMKALVLFKPE-------TRGLKDPEHVEALQDQSQ  350
                  H7            H8                                H9

              *  : ::::* ** *** *** **:** :     *  :** : :*: *:  *: **
NR2F6        VALTEYVRAQYPSQPQRFGRLLLRLPALRAVPASLISQLFFMRLVGKTPIETLIRDMLLS  392
COUP-TF2     CALEEYVRSQYPNQPTRFGKLLLRLPSLRTVSSSVIEQLFFVRLVGKTPIETLIRDMLLS  402
TLX          LTLNSYIHTRYPTQPCRFGKLLLLLPALRSISPSTIEEVFFKKTIGNVPITRLLSDMYKS  382
PNR          VMLSQHSKAHHPSQPVRFGKLLLLLPSLRFITAERIELLFFRKTIGNTPMEKLLCDMFKN  410
                                  H10                           H12

              ▼
NR2F6        GSTFNWPYGSGQ         404
COUP-TF2     GSSFNWPYMAIQ         414
TLX          SDI---------         385
PNR          ------------
```

B

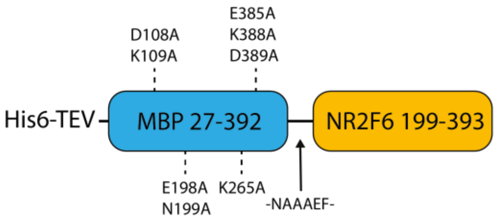

C

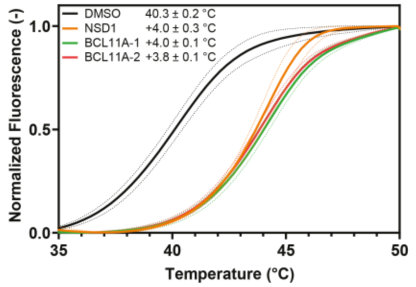

Figure S2: Design and characterization of the MBP-NR2F6 fusion construct. A. Sequence alignment of the NR2F6 LBD with the LBDs of COUP-TF2, TLX and PNR NRs. Bars represent predicted alpha helices of NR2F6. Identical residues are indicated with an asterisk (*). Residues with similar chemical properties are indicated with a colon (:). Black triangles indicate start and end of the NR2F6 segment fused to MBP. **B.** Design of MBP-NR2F6 fusion construct with surface entropy reduction mutations. **C.** DSF curves of MBP-NR2F6 in the presence of DMSO or NSD1, BCL11A-1 or BCL11A-2 peptide. Data shown is the average and standard deviation of two independent experiments.

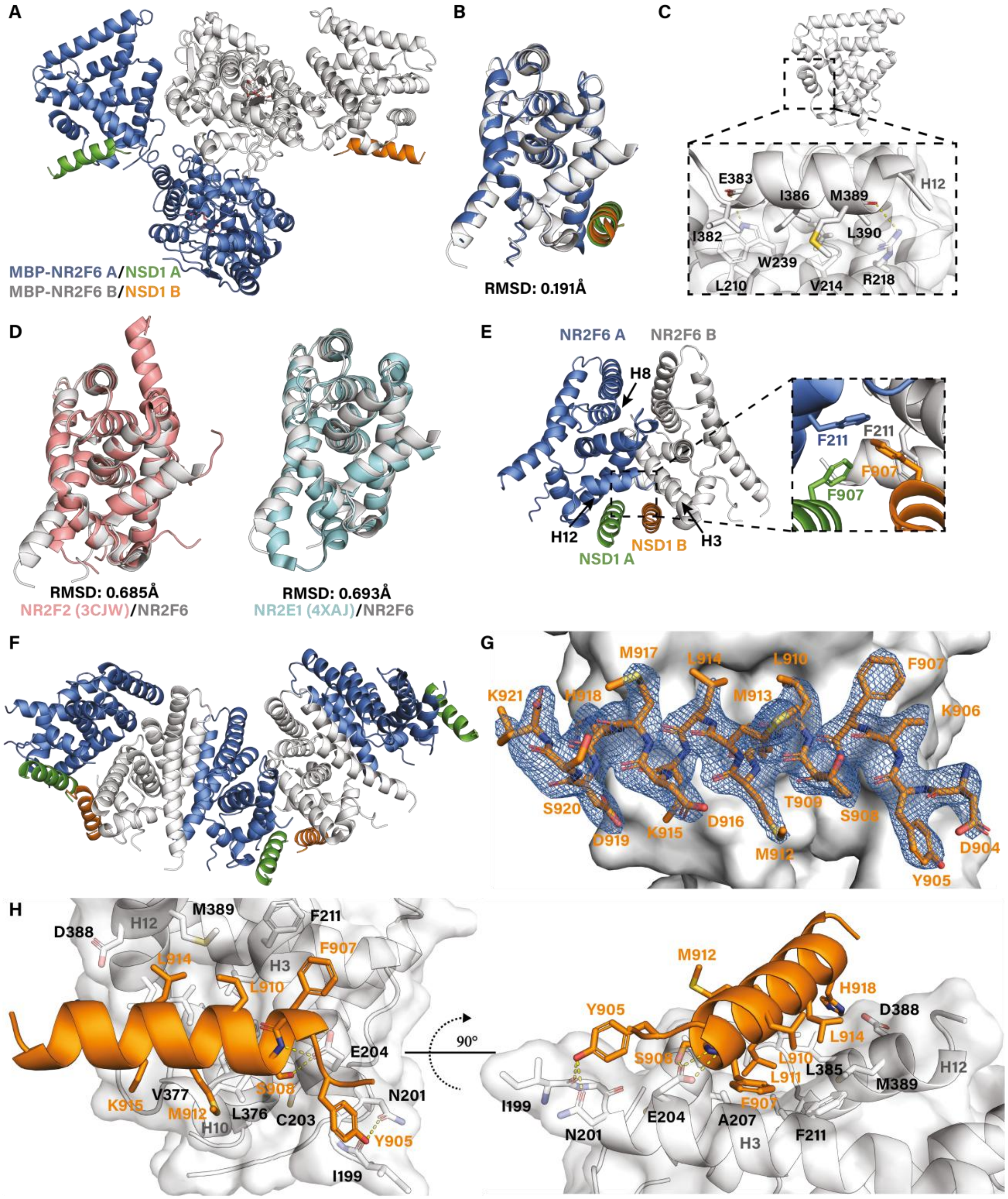

Figure S3: Additional NR2F6 crystal structure details. A. Crystallographic asymmetric unit. **B.** Structural alignment of both NR2F6/NSD1 complexes present in the asymmetric unit. **C.** Zoom-in of H12 bound to the canonical coregulator binding site. **D.** Structural alignment of the NR2F6 LBD with COUP-TF2 (NR2F2, PDB: 3CJW) and TLX (NR2E1, PDB: 4XAJ). **E.** Second dimerization interface present in

the crystal structure. **F.** Pentamer of NR2F6 LBDs formed through both dimerization interfaces. **G.** 2Fo-Fc electron density map (blue mesh) of NSD1 of monomer A contoured at 1.0 σ. **H.** Zoom-in of the NR2F6/NSD1 interaction of monomer A.

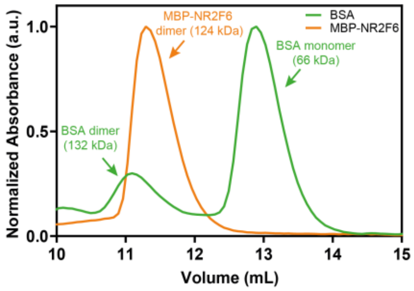

Figure S4: Analytical SEC chromatogram of MBP-NR2F6 and BSA.

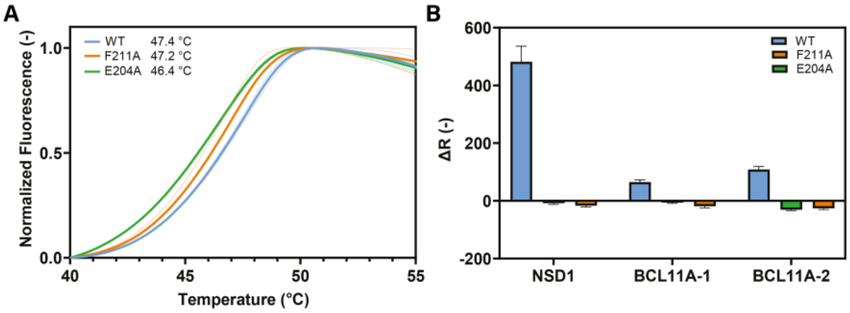

Figure S5: NR2F6^{E204A} and NR2F6^{F211A} DSF and TR-FRET characterization. A. DSF curves of wildtype NR2F6 LBD, NR2F6^{F211A} LBD and NR2F6^{E204A} LBD, data in technical triplicate. **B.** TR-FRET recruitment of biotinylated NSD1, BCL11A-1 and BCL11A-2 coregulator peptides to NR2F6 LBD, NR2F6^{F211A} LBD and NR2F6^{E204A} LBD. Data shown is the average and standard deviation of three independent experiments.

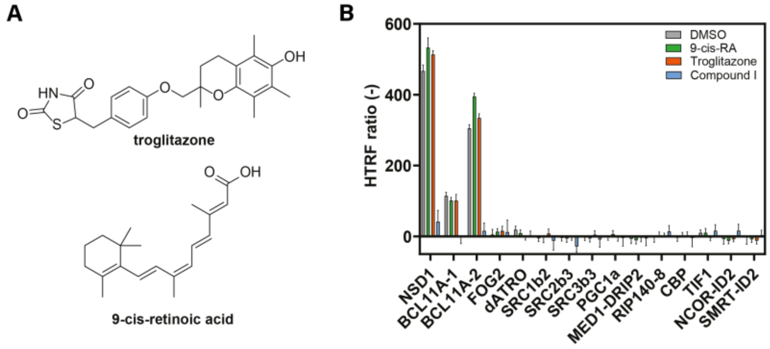

Figure S6: Profiling of proposed NR2F6 LBD binders. A. Chemical structures of troglitazone and 9-cis-retinoic acid. **B.** TR-FRET recruitment of NR coregulator peptides by NR2F6 in the presence of 50 µM troglitazone or 9-cis-retinoic acid, or when covalently bound to compound **I**. Data in technical triplicate.

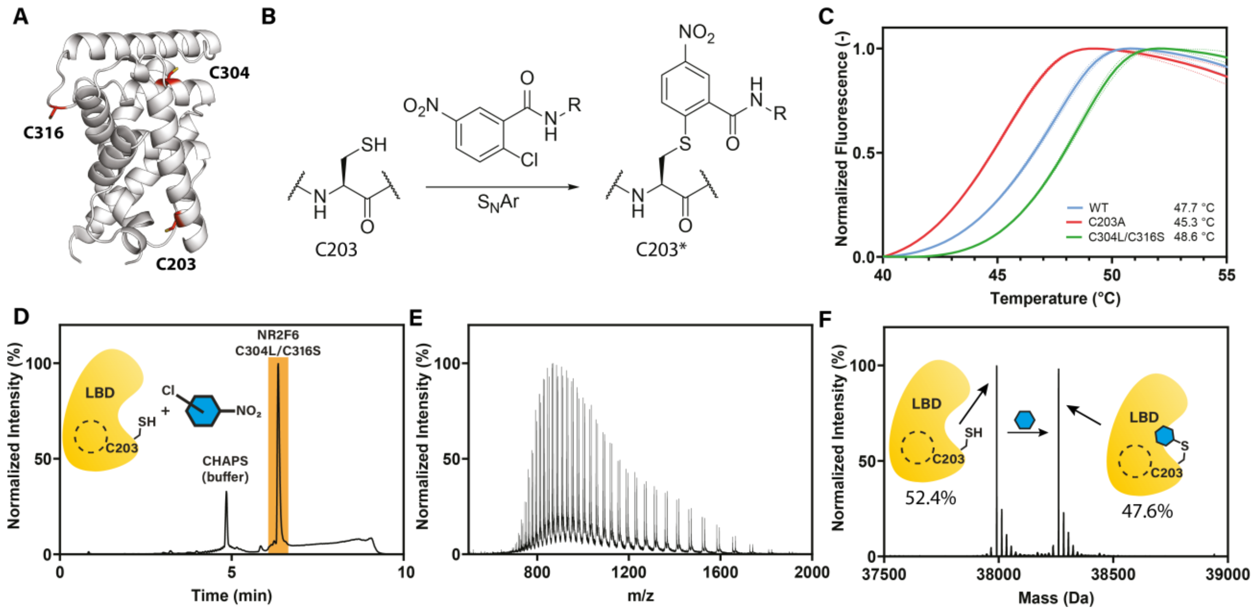

Figure S7: NR2F6 cysteine mutants and covalent probe screening. A. NR2F6 LBD with its three cysteine residues highlighted in red. **B.** Covalent labeling of C203 of NR2F6 by electron-deficient haloarenes via nucleophilic aromatic substitution. **C.** DSF curves of wildtype NR2F6 LBD, NR2F6^{C203A} LBD and NR2F6$^{C304L/C316S}$ LBD, data in technical triplicate. **D.** Representative chromatogram of the covalent probe screening. The area highlighted in orange corresponds to the protein peak analyzed for each library member. **E.** Representative *m/z* spectrum, containing both the *m/z* envelope of unbound and bound NR2F6 LBD. **F.** Deconvoluted spectrum of **E**, revealing the presence of both labeled and unlabeled NR2F6. Degree of labeling is calculated from the peak intensities.

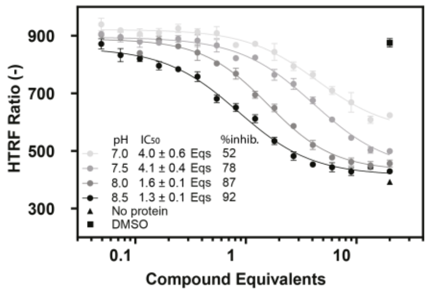

Figure S8: pH dependency of compound I. Replicate data of pH-dependent displacement of bio-NSD1 in TR-FRET by **I**. Data recorded in triplicate and presented as the average ± SD.

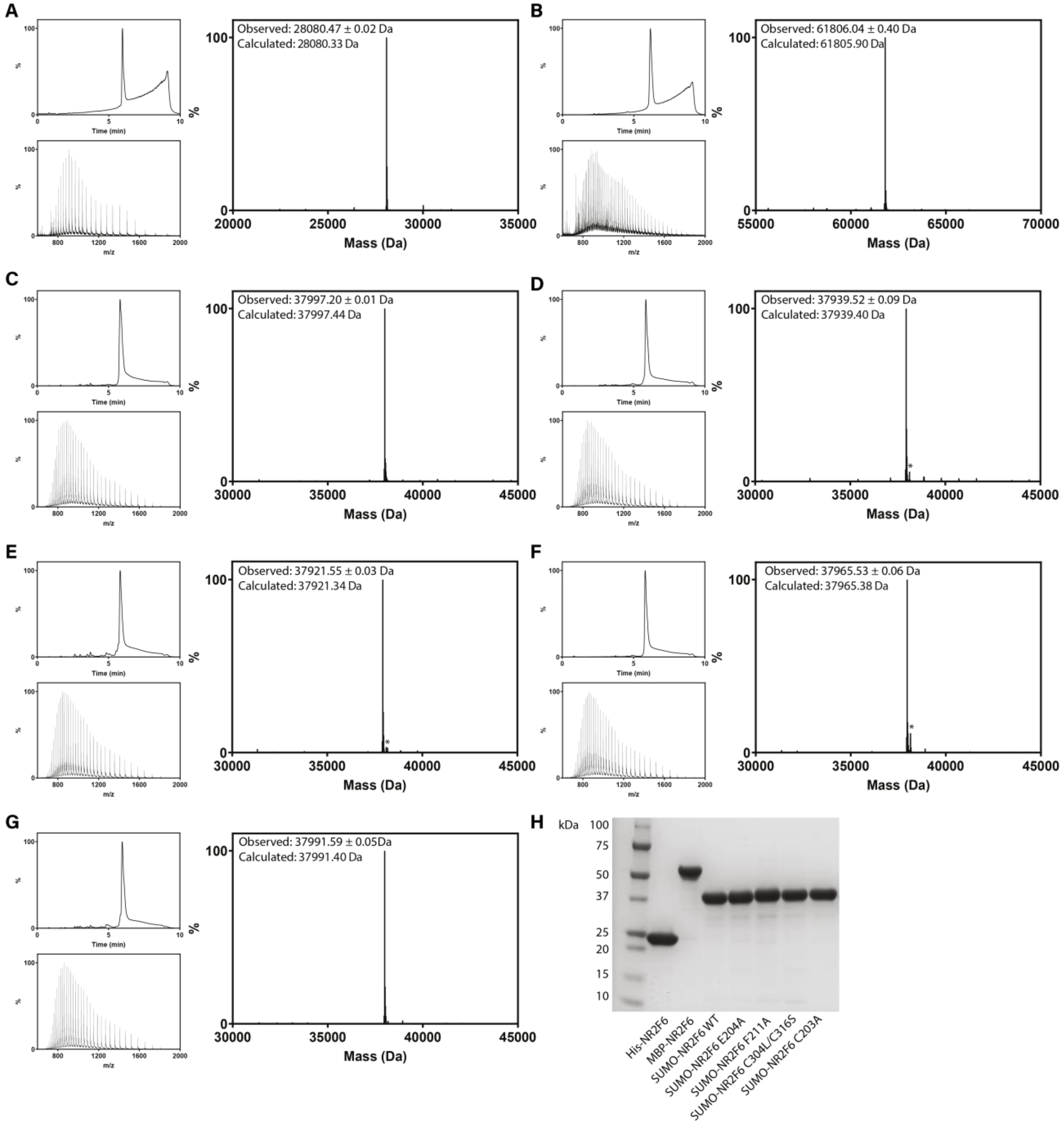

Figure S9: Protein Q-ToF MS and SDS-Page. Q-ToF MS spectra of His-NR2F6 LBD (**A**), MBP-NR2F6 LBD (**B**), His-SUMO-NR2F6 LBD (**C**), His-SUMO-NR2F6 LBD E204A (**D**), His-SUMO-NR2F6 LBD F211A (**E**), His-SUMO-NR2F6 LBD C203A (**F**), His-SUMO-NR2F6 LBD C304L/C316S (**G**). Asterisk (*) indicates alpha-N-6-phosphogluconoylation of His-tag (+178 Da).[26] **H.** SDS-Page of all protein constructs used in this study.

Table S1: Peptide sequences

Peptide	Sequence
**bio-SRC1b2**	biotin-Ahx-PSSHSSLTERHKILHRLLQEGSPSD-NH2
**bio-SRC2b3**	biotin-Ahx-PKKKENALLRYLLDKDDTKDI-NH2
**bio-SRC3b3**	biotin-Ahx-PKKENNALLRYLLDRDDPSDV-NH2
**bio-PGC-1α**	biotin-Ahx-DGTPPPQEAEEPSLLKKLLLAPANT-NH2
**bio-MED1-DRIP2**	biotin-Ahx-VSSMAGNTKNHPMLMNLLKDNPAQ-NH2
**bio-RIP140-8**	biotin-Ahx-FSFSKNGLLSRLLRQNQDSYL-NH2
**bio-CBP**	biotin-Ahx-DAASKHKQLSELLRGGSGSSI-NH2
**bio-TIF-1**	biotin-Ahx-NANYPRSILTSLLLNSSQSST-NH2
**bio-NCOR-ID2**	biotin-Ahx-ADPASNLGLEDIIRKALMGSF-NH2
**bio-SMRT-ID2**	biotin-Ahx-AVQEHASTNMGLEAIIRKALMGKYD-NH2
**bio-dATRO**	biotin-Ahx-PPYADTPALRQLSEYARPHVAFSP-NH2
**bio-NSD1**	biotin-Ahx-ASSQNHIPIEPDYKFSTLLMMLKDMHDSKT-NH2
**bio-BCL11A-1**	biotin-Ahx-LRLNPMAMEPPAMDFSRRLRELAGNTSSP-NH2
**bio-BCL11A-2**	biotin-Ahx-PPAAMPNTENVYSQWLAGYAASRQLKDP-NH2
**bio-FOG2**	biotin-Ahx-KNGNLKQPSPNGNLFSSHLATLQGLKVFSE-NH2
**ac-dATRO**	Ac- PPYADTPALRQLSEYARPHVAFSP-NH2
**ac-NSD1**	Ac-DYKFSTLLMMLKDMHDSKT-NH2
**ac-BCL11A-1**	Ac-AMDFSRRLRELAGNTSSPP-NH2
**ac-BCL11A-2**	Ac-ENVYSQWLAGYAASRQLKD-NH2
**ac-FOG2**	Ac-GNLFSSHLATLQGLKVFSE-NH2
**FAM-NSD1**	5-FAM-Ahx-DYKFSTLLMMLKDMHDSKT-NH2

Table S2: Data collection and refinement statistics

PDB	8C5L
**Protein**	MBP-NR2F6
**Peptide**	NSD1
**Beamline**	ESRF ID23-1
Data collection	
**Wavelength (Å)**	0.972425
**Space group**	P 61
**Cell dimensions**	
**a, b, c (Å)**	170.38, 170.38, 83.67
**α, β, γ (°)**	90, 90, 120
**Resolution (Å)**	55.34-2.60 (2.69-2.60)
**$I / \sigma(I)$**	11.7 (1.2)
**Completeness (%)**	100.0 (100)
**Redundancy**	9.6 (10.1)
**$CC_{1/2}$**	0.999 (0.376)
Refinement	
**No. reflections**	42757 (4485)
**R_{work}/R_{free}**	0.200 / 0.233
**No. atoms (non-H)**	
**Protein**	8361
**Peptide**	289
**Ligand**	46
**Solvent**	56
**Average B-factors**	73.0
**R.m.s. deviations**	
**Bond lengths (Å)**	0.006
**Bond angles (°)**	0.895
**Ramachandran**	
**favored (%)**	97.27
**outliers (%)**	0.00

Table S3: Covalent library screening results

Compound	%C304L/C316S	%C203A	Compound	%C304L /C316S	%C203A
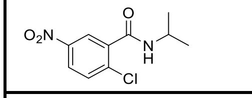	0	0	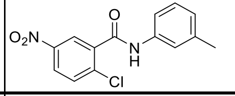	56	5
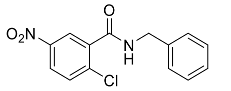	0	0	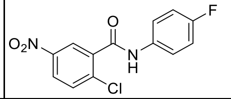	57	14
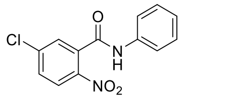	0	0	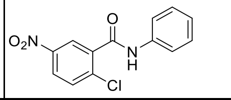	62	20
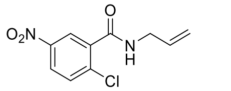	3	0	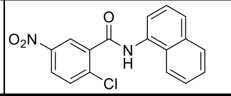	65	4
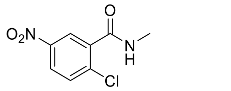	3	0	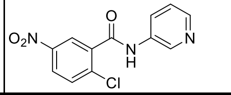	66	17
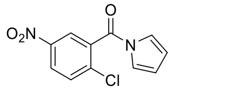	7	0	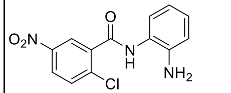	77	7
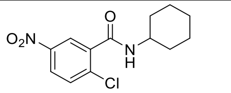	8	0	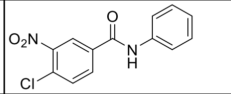	80	62
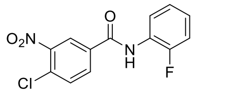	18	7	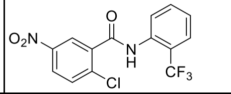	82	17
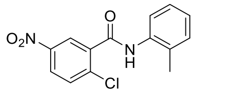	20	0	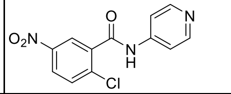	82	24
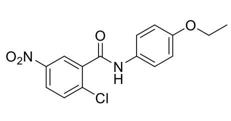	20	0	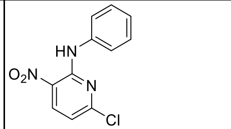	87	28
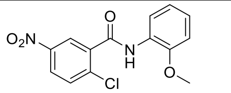	26	0	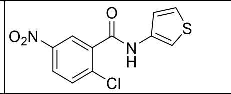	88	15
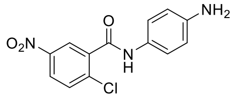	41	0	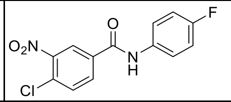	90	67
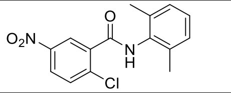	42	3	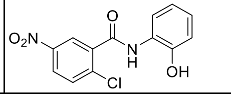	90	20
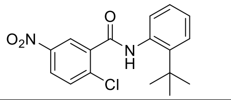	43	5	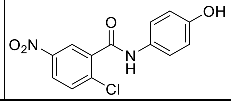	92	10
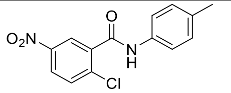	47	0	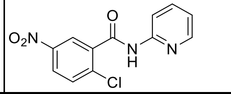	94	19
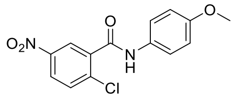	48	0	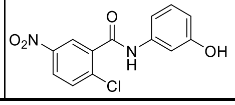	95	34

Table S3: Covalent library screening results (continued)

Compound	%C304L/C316S	%C203A	Compound	%C304L /C316S	%C203A
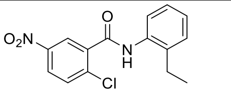	**48**	**0**	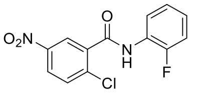	**98**	**32**
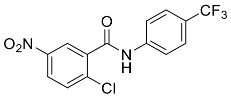	**54**	**0**	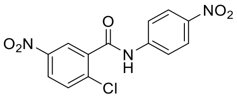	**100**	**100**

NMR spectra (^{1}H & ^{13}C) and LC-UV traces for assayed compounds

4-chloro-3-nitro-N-phenylbenzamide

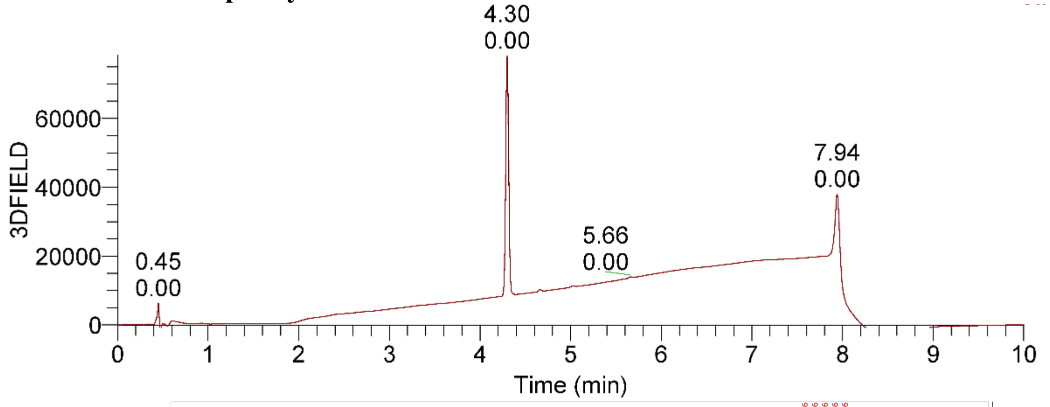

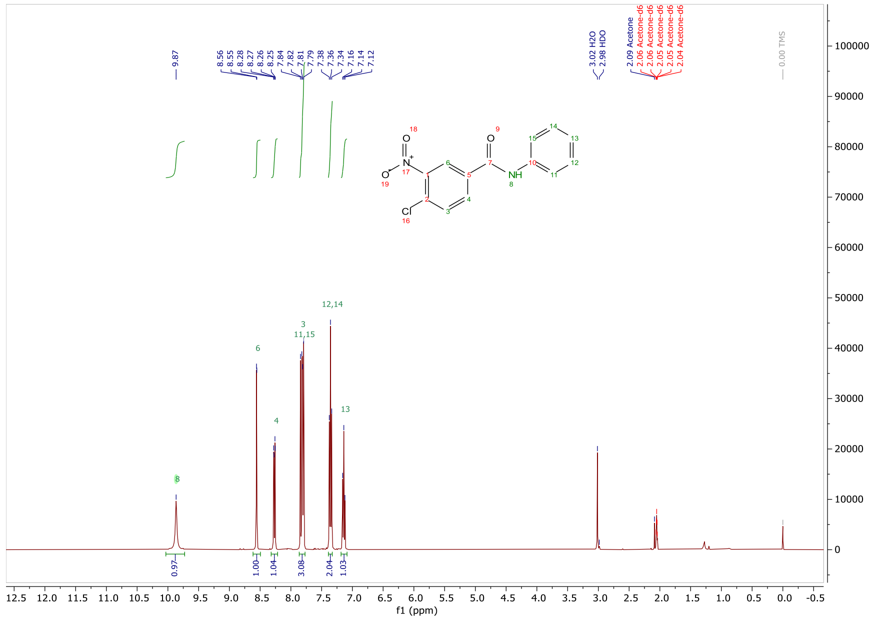

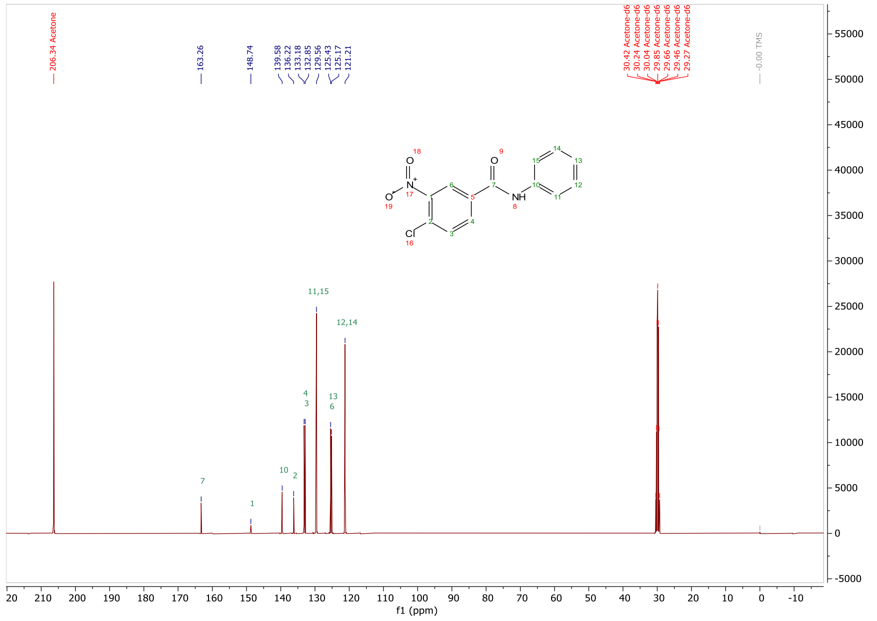

2-chloro-N-(4-fluorophenyl)-5-nitrobenzamide

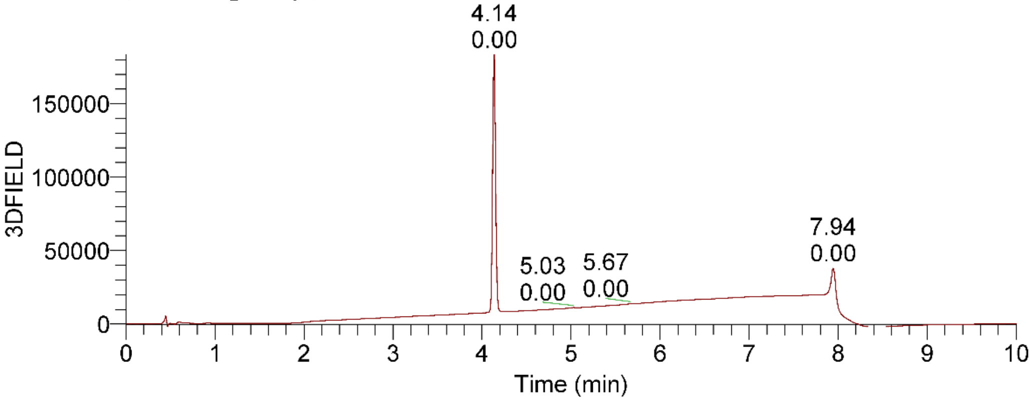

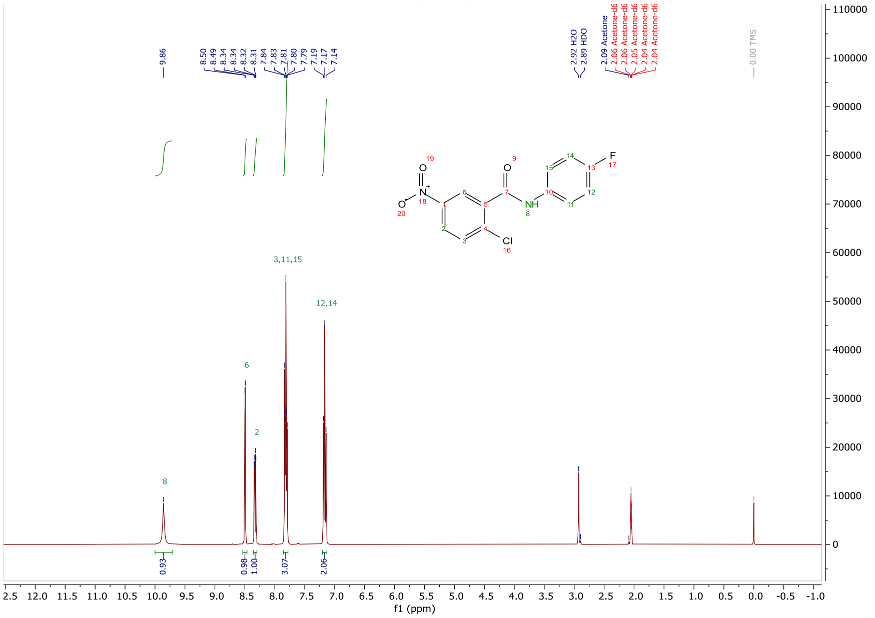

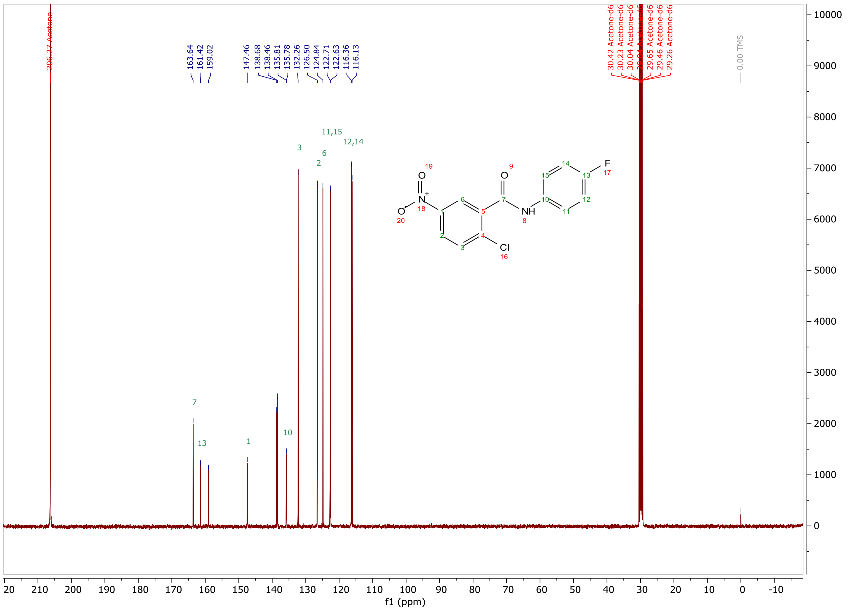

4-chloro-N-(2-fluorophenyl)-3-nitrobenzamide

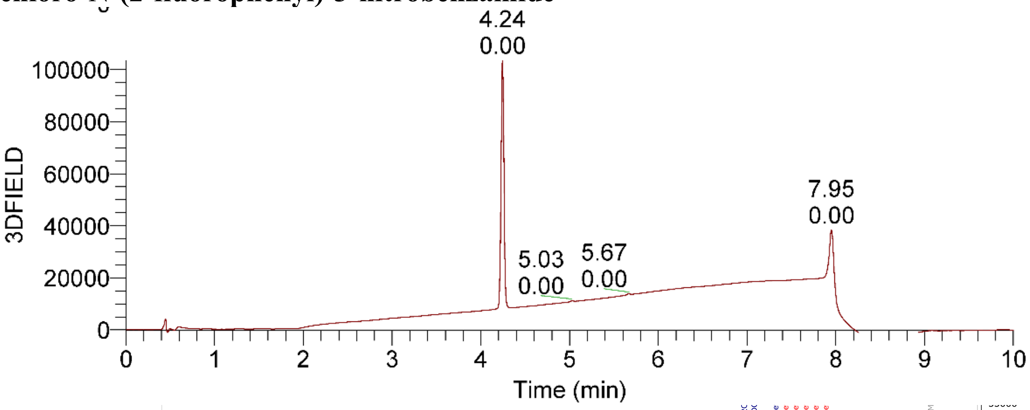

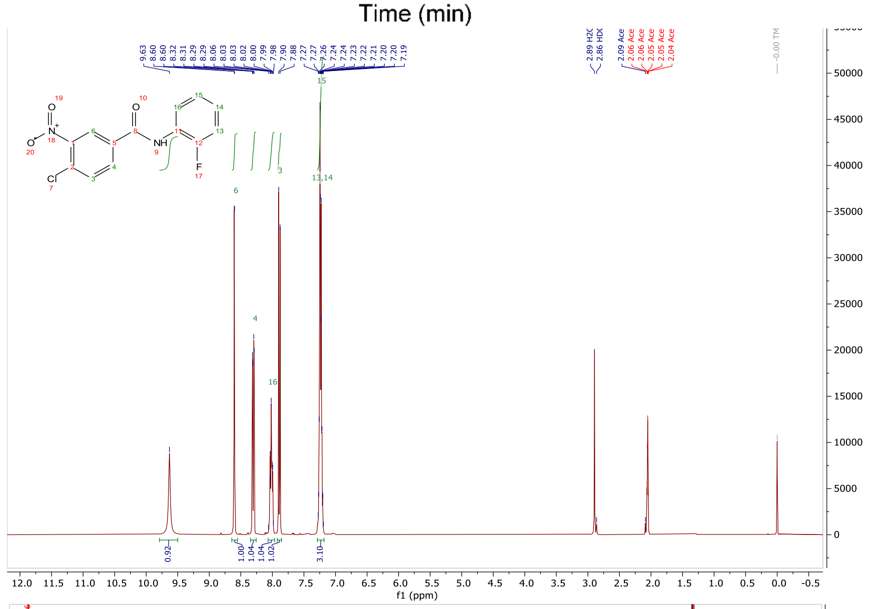

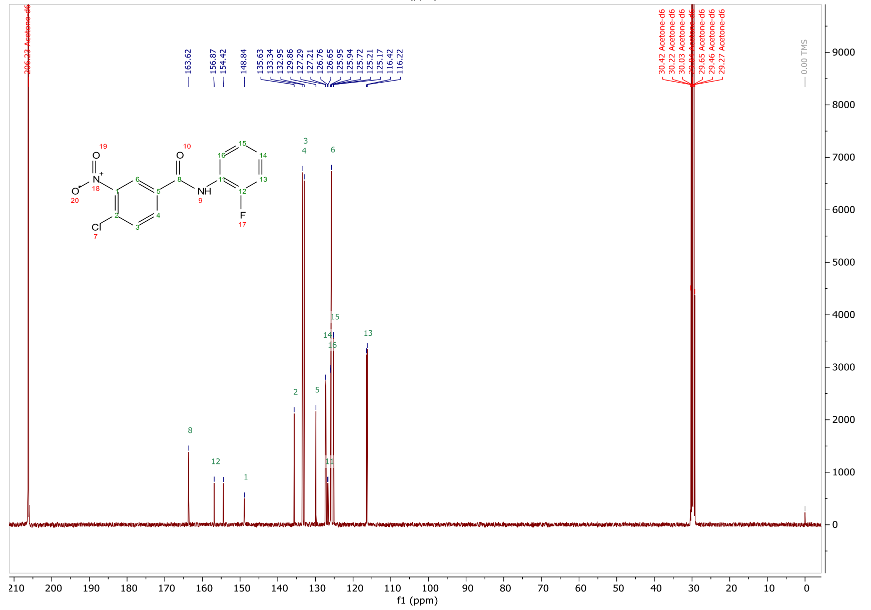

4-chloro-N-(4-fluorophenyl)-3-nitrobenzamide

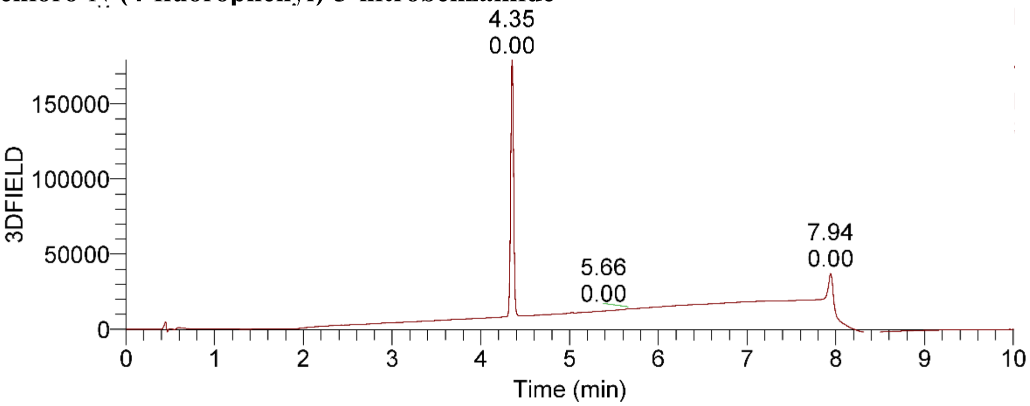

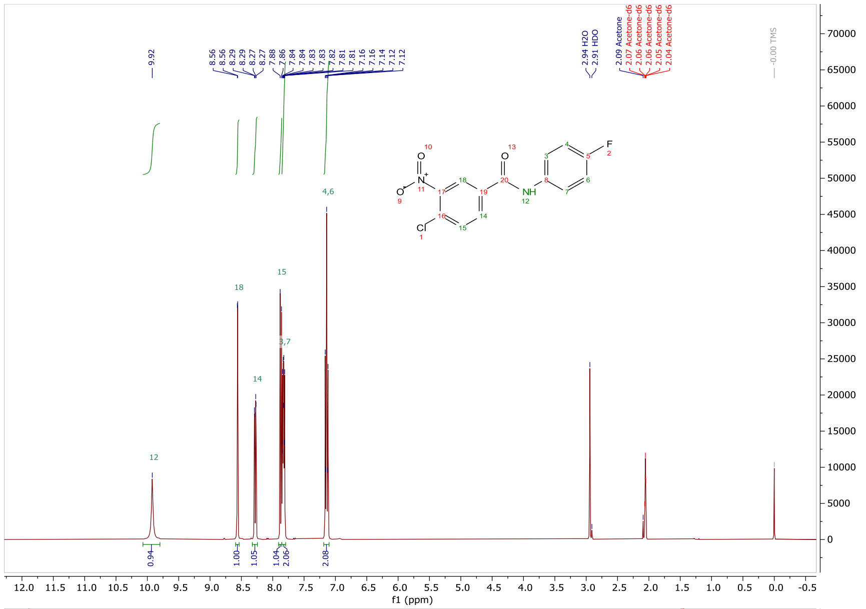

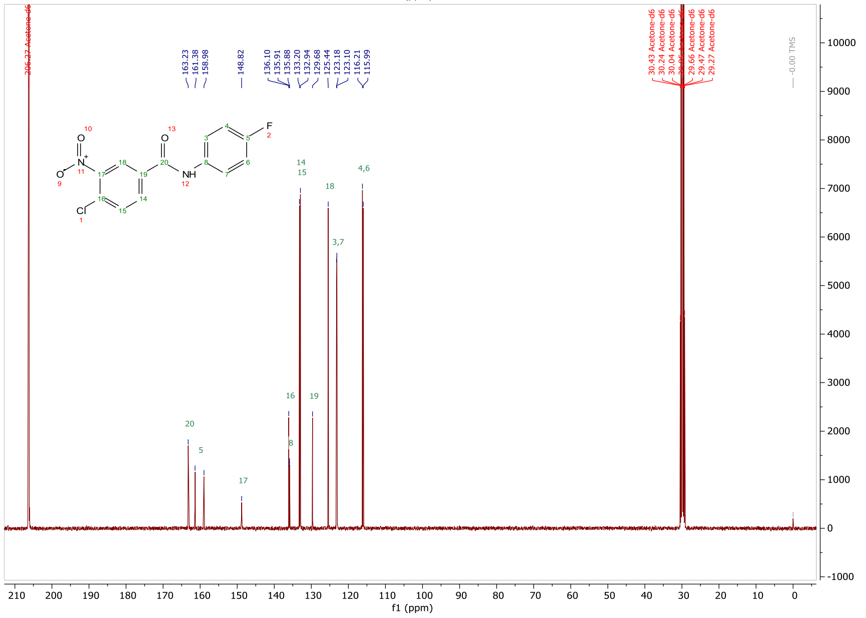

2-chloro-5-nitro-N-(4-(trifluoromethyl)phenyl)benzamide

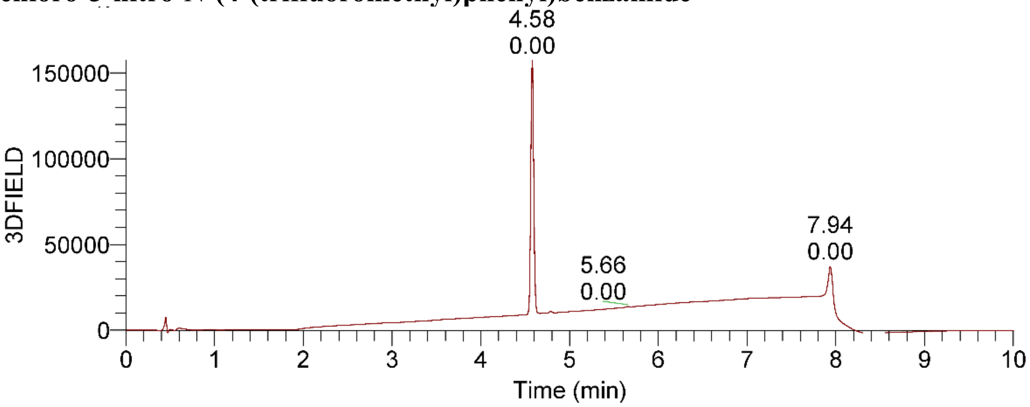

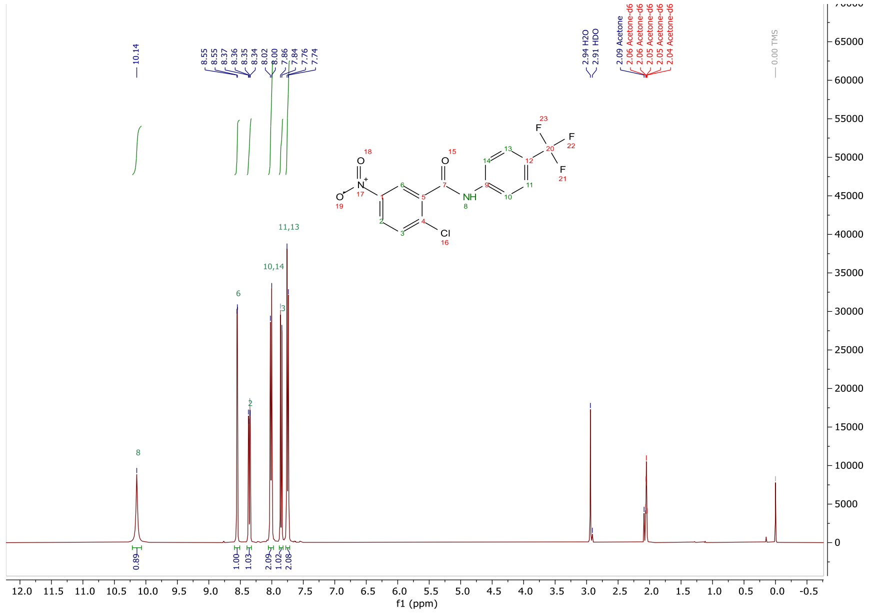

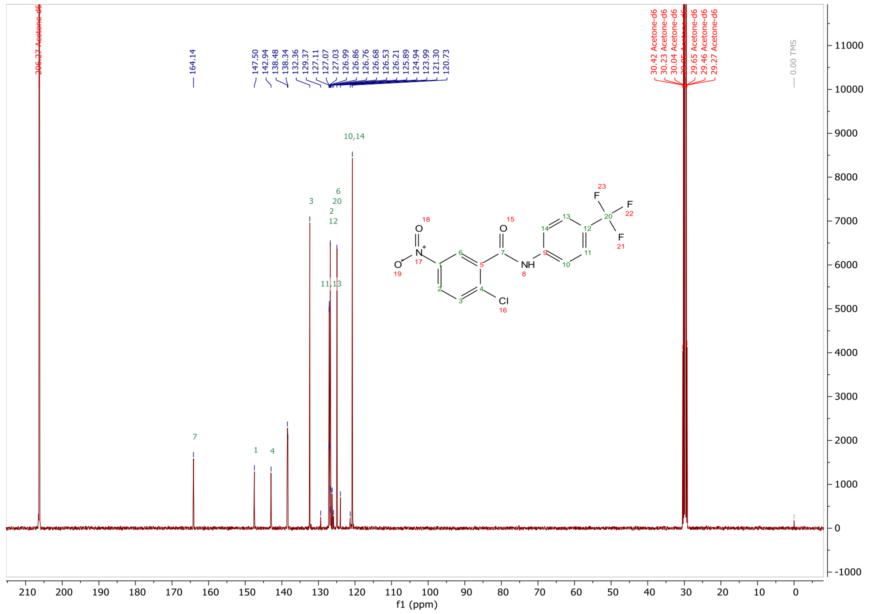

2-chloro-N-(4-ethoxyphenyl)-5-nitrobenzamide

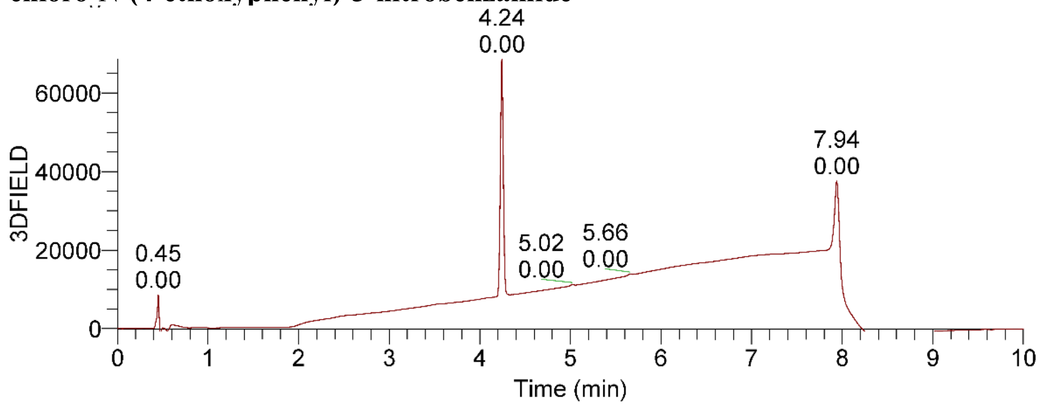

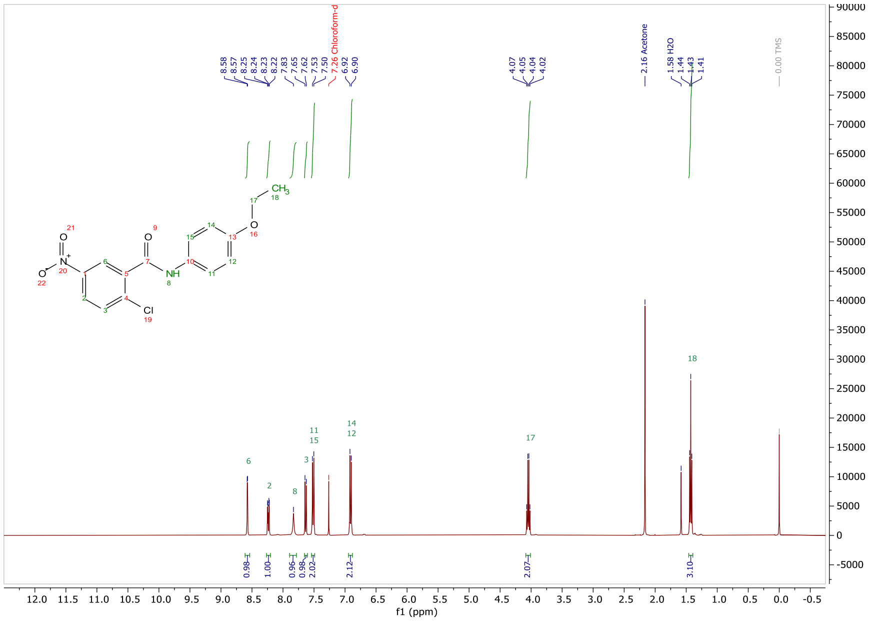

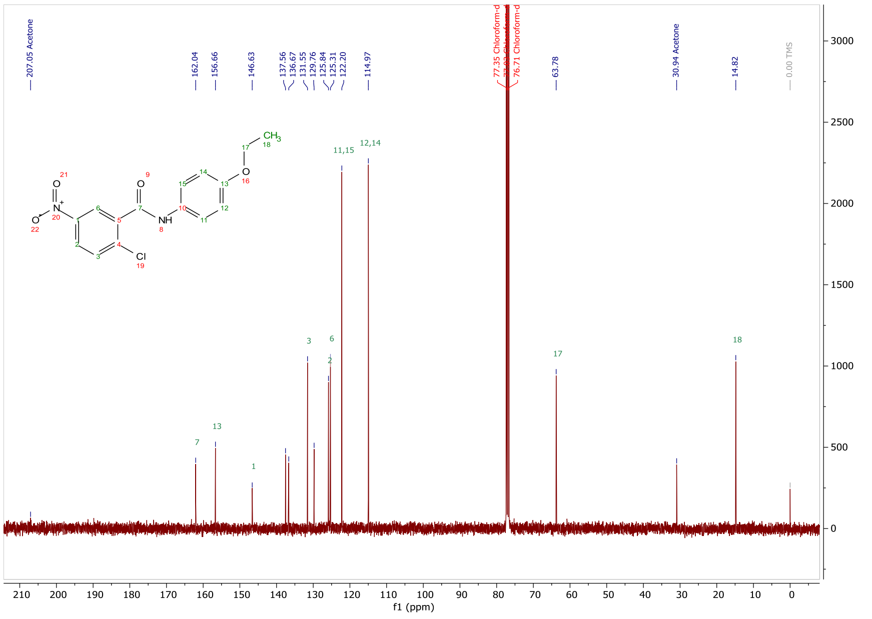

2-chloro-N-(3-hydroxyphenyl)-5-nitrobenzamide

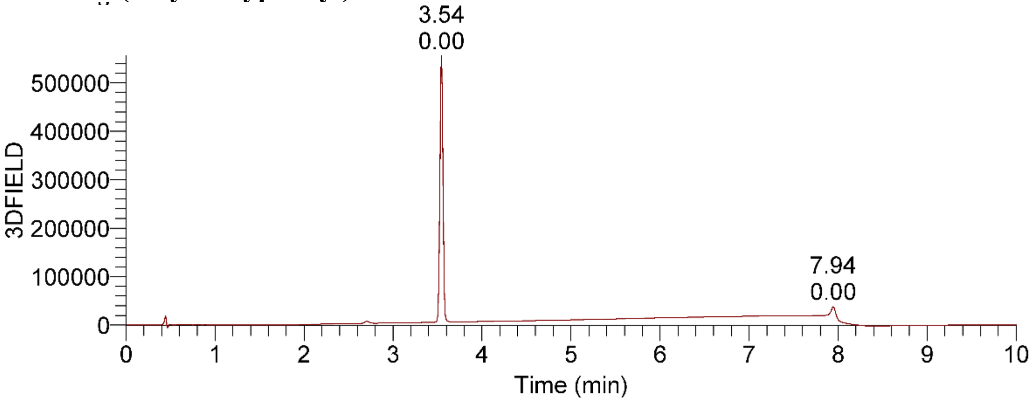

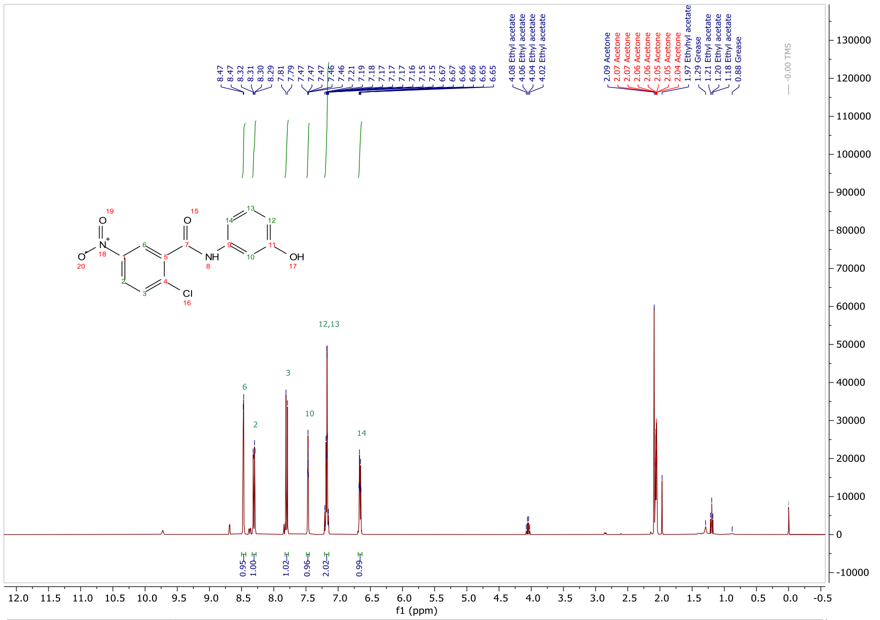

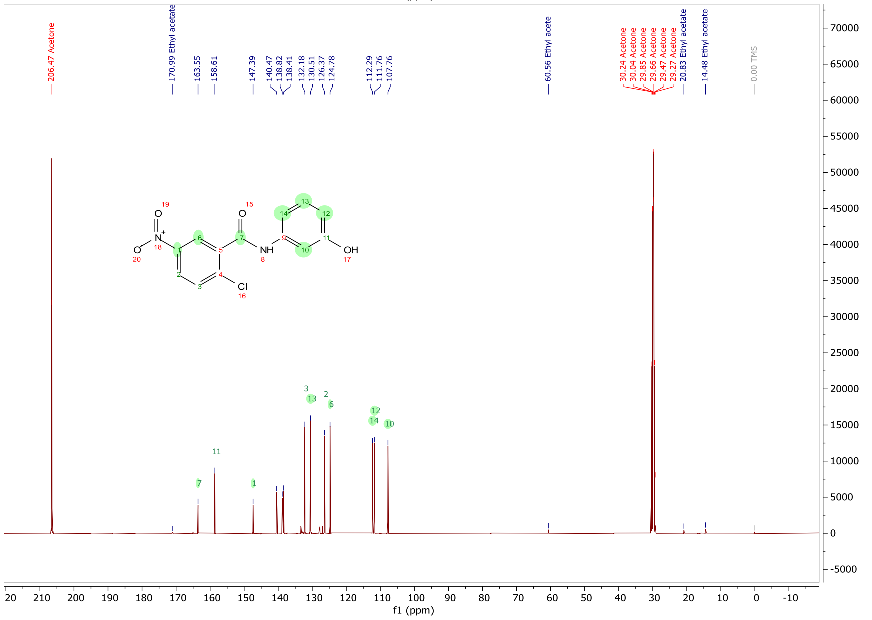

2-chloro-N-(4-hydroxyphenyl)-5-nitrobenzamide

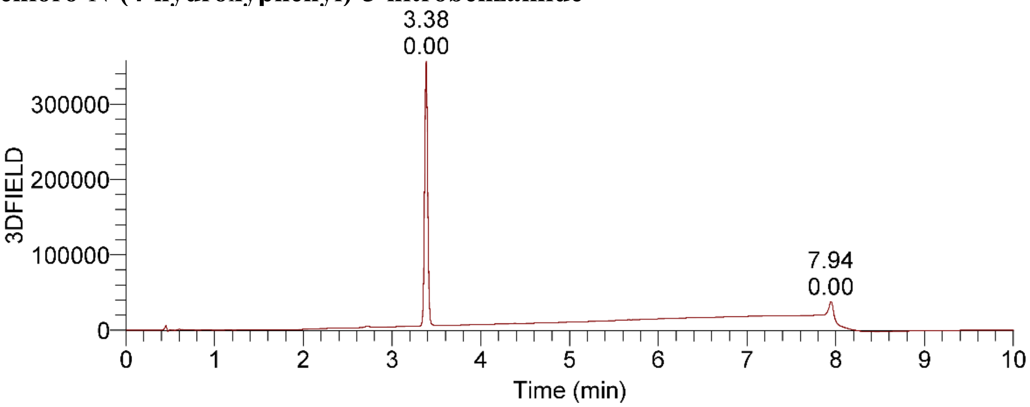

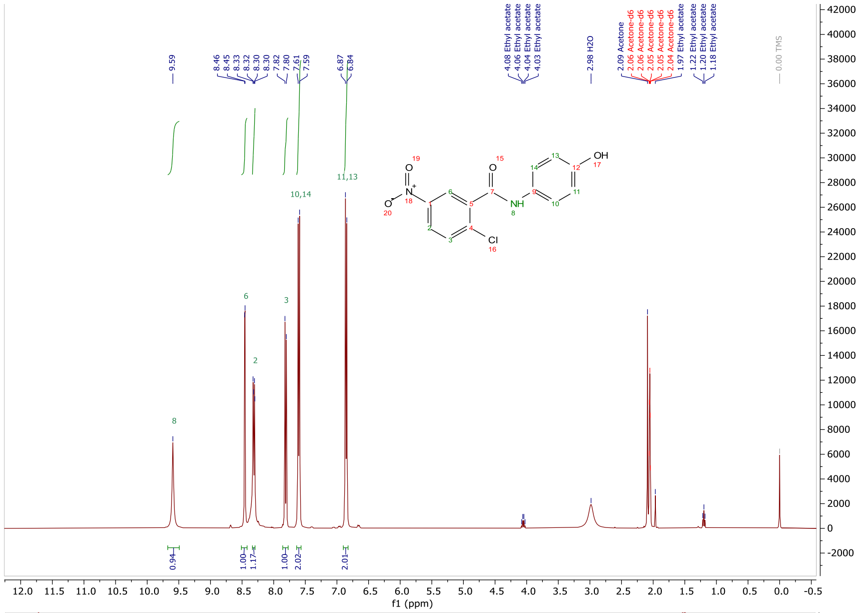

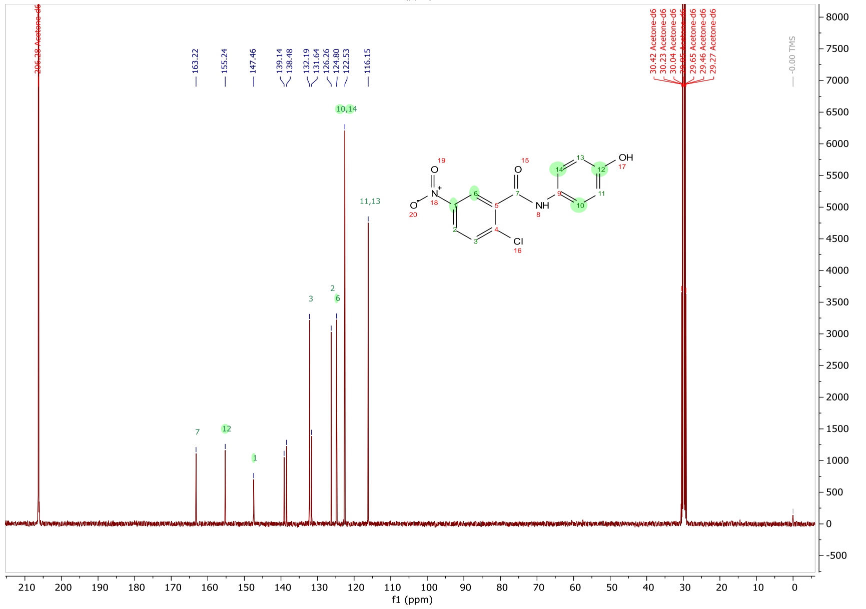

N-(4-aminophenyl)-2-chloro-5-nitrobenzamide

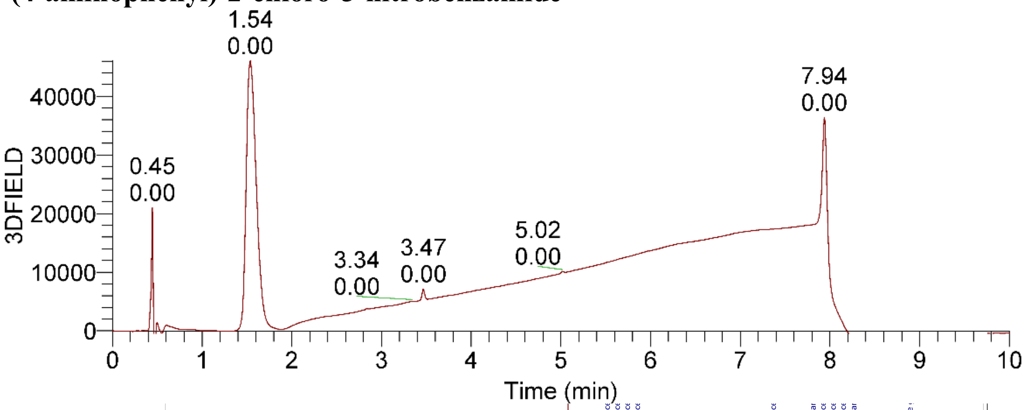

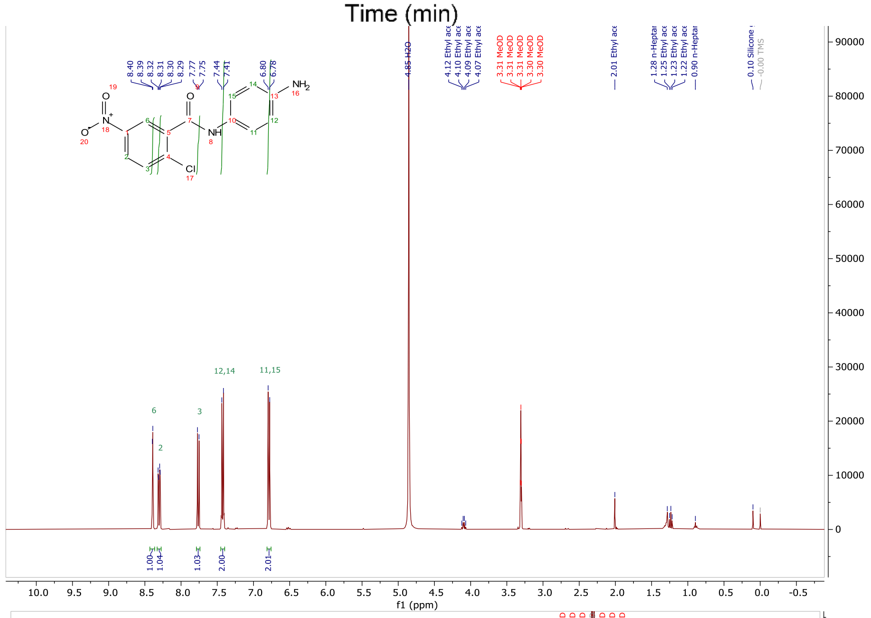

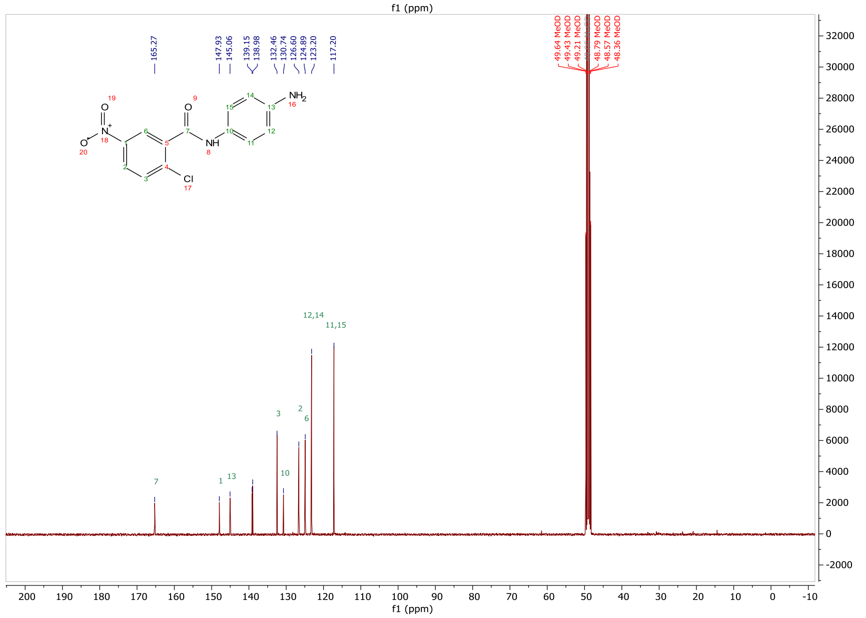

6-chloro-3-nitro-N-phenylpyridin-2-amine

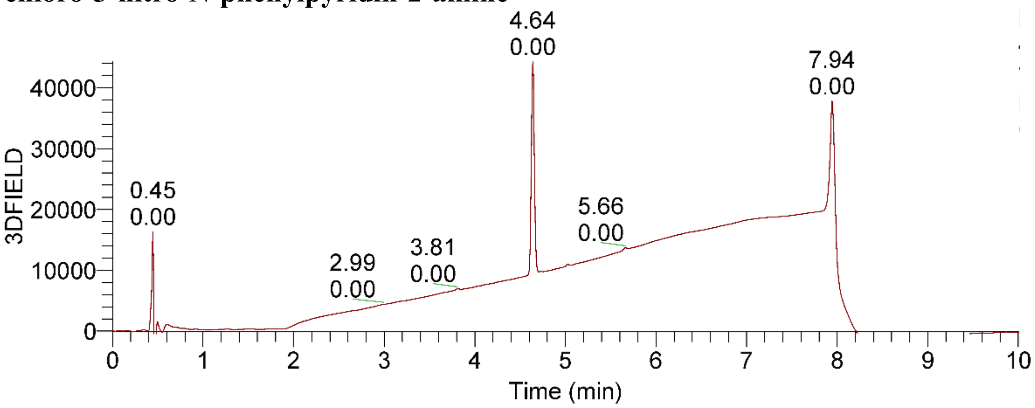

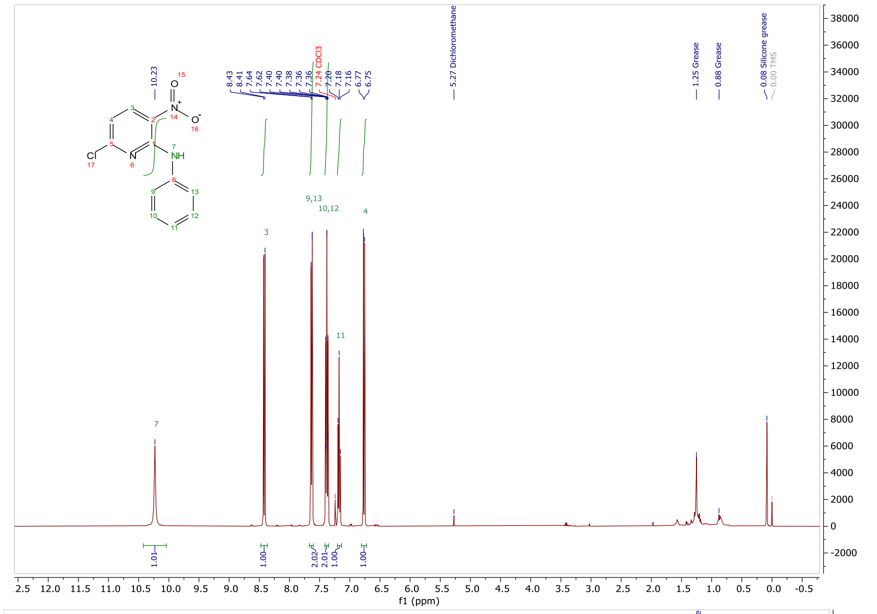

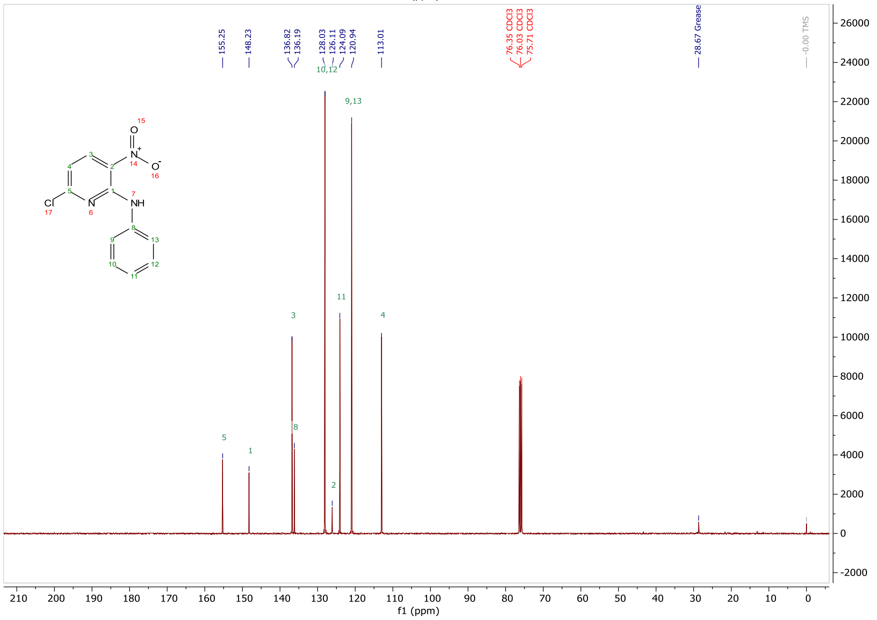

4. Supporting References

(1) Waterhouse, A.; Bertoni, M.; Bienert, S.; Studer, G.; Tauriello, G.; Gumienny, R.; Heer, F. T.; De Beer, T. A. P.; Rempfer, C.; Bordoli, L.; Lepore, R.; Schwede, T. SWISS-MODEL: Homology Modelling of Protein Structures and Complexes. *Nucleic Acids Res* **2018**, *46* (W1), W296–W303. https://doi.org/10.1093/NAR/GKY427.

(2) Mariani, V.; Biasini, M.; Barbato, A.; Schwede, T. LDDT: A Local Superposition-Free Score for Comparing Protein Structures and Models Using Distance Difference Tests. *Bioinformatics* **2013**, *29* (21), 2722–2728. https://doi.org/10.1093/BIOINFORMATICS/BTT473.

(3) Bertoni, M.; Kiefer, F.; Biasini, M.; Bordoli, L.; Schwede, T. Modeling Protein Quaternary Structure of Homo- and Hetero-Oligomers beyond Binary Interactions by Homology. *Sci Rep* **2017**, *7* (1). https://doi.org/10.1038/S41598-017-09654-8.

(4) Benkert, P.; Biasini, M.; Schwede, T. Toward the Estimation of the Absolute Quality of Individual Protein Structure Models. *Bioinformatics* **2011**, *27* (3), 343–350. https://doi.org/10.1093/BIOINFORMATICS/BTQ662.

(5) Studer, G.; Biasini, M.; Schwede, T. Assessing the Local Structural Quality of Transmembrane Protein Models Using Statistical Potentials (QMEANBrane). *Bioinformatics* **2014**, *30* (17). https://doi.org/10.1093/BIOINFORMATICS/BTU457.

(6) Studer, G.; Rempfer, C.; Waterhouse, A. M.; Gumienny, R.; Haas, J.; Schwede, T. QMEANDisCo-Distance Constraints Applied on Model Quality Estimation. *Bioinformatics* **2020**, *36* (6), 1765–1771. https://doi.org/10.1093/BIOINFORMATICS/BTZ828.

(7) Studer, G.; Tauriello, G.; Bienert, S.; Biasini, M.; Johner, N.; Schwede, T. ProMod3-A Versatile Homology Modelling Toolbox. *PLoS Comput Biol* **2021**, *17* (1). https://doi.org/10.1371/JOURNAL.PCBI.1008667.

(8) Guex, N.; Peitsch, M. C.; Schwede, T. Automated Comparative Protein Structure Modeling with SWISS-MODEL and Swiss-PdbViewer: A Historical Perspective. *Electrophoresis* **2009**, *30 Suppl 1* (SUPPL. 1). https://doi.org/10.1002/ELPS.200900140.

(9) Waterhouse, A. M.; Studer, G.; Robin, X.; Bienert, S.; Tauriello, G.; Schwede, T. The Structure Assessment Web Server: For Proteins, Complexes and More. *Nucleic Acids Res* **2024**, *52* (W1), W318–W323. https://doi.org/10.1093/NAR/GKAE270.

(10) Bienert, S.; Waterhouse, A.; De Beer, T. A. P.; Tauriello, G.; Studer, G.; Bordoli, L.; Schwede, T. The SWISS-MODEL Repository-New Features and Functionality. *Nucleic Acids Res* **2017**, *45* (D1), D313–D319. https://doi.org/10.1093/NAR/GKW1132.

(11) Le Guilloux, V.; Schmidtke, P.; Tuffery, P. Fpocket: An Open Source Platform for Ligand Pocket Detection. *BMC Bioinformatics* **2009**, *10* (1), 1–11. https://doi.org/10.1186/1471-2105-10-168/TABLES/1.

(12) Meijer, F. A.; Van Den Oetelaar, M. C. M.; Doveston, R. G.; Sampers, E. N. R.; Brunsveld, L. Covalent Occlusion of the RORγt Ligand Binding Pocket Allows Unambiguous Targeting of an Allosteric Site. *ACS Med Chem Lett* **2021**, *12*, 25. https://doi.org/10.1021/acsmedchemlett.1c00029.

(13) Vonrhein, C.; Flensburg, C.; Keller, P.; Sharff, A.; Smart, O.; Paciorek, W.; Womack, T.; Bricogne, G. Data Processing and Analysis with the AutoPROC Toolbox. *urn:issn:0907-4449* **2011**, *67* (4), 293–302. https://doi.org/10.1107/S0907444911007773.

(14) Evans, P. R. An Introduction to Data Reduction: Space-Group Determination, Scaling and Intensity Statistics. *Acta Crystallogr D Biol Crystallogr* **2011**, *67* (Pt 4), 282–292. https://doi.org/10.1107/S090744491003982X.

(15) Evans, P. R.; Murshudov, G. N. How Good Are My Data and What Is the Resolution? *Acta Crystallogr D Biol Crystallogr* **2013**, *69* (Pt 7), 1204. https://doi.org/10.1107/S0907444913000061.

(16) Potterton, L.; Agirre, J.; Ballard, C.; Cowtan, K.; Dodson, E.; Evans, P. R.; Jenkins, H. T.; Keegan, R.; Krissinel, E.; Stevenson, K.; Lebedev, A.; McNicholas, S. J.; Nicholls, R. A.; Noble, M.; Pannu, N. S.; Roth, C.; Sheldrick, G.; Skubak, P.; Turkenburg, J.; Uski, V.; Von Delft, F.; Waterman, D.; Wilson, K.; Winn, M.; Wojdyr, M. CCP4i2: The New Graphical User Interface to the CCP4 Program Suite. *Acta Crystallogr D Struct Biol* **2018**, *74* (Pt 2), 68–84. https://doi.org/10.1107/S2059798317016035.

(17) McCoy, A. J. Solving Structures of Protein Complexes by Molecular Replacement with Phaser. *Acta Crystallogr D Biol Crystallogr* **2007**, *63* (Pt 1), 32–41. https://doi.org/10.1107/S0907444906045975.

(18) Mirdita, M.; Schütze, K.; Moriwaki, Y.; Heo, L.; Ovchinnikov, S.; Steinegger, M. ColabFold: Making Protein Folding Accessible to All. *Nature Methods 2022 19:6* **2022**, *19* (6), 679–682. https://doi.org/10.1038/s41592-022-01488-1.

(19) Jumper, J.; Evans, R.; Pritzel, A.; Green, T.; Figurnov, M.; Ronneberger, O.; Tunyasuvunakool, K.; Bates, R.; Žídek, A.; Potapenko, A.; Bridgland, A.; Meyer, C.; Kohl, S. A. A.; Ballard, A. J.; Cowie, A.; Romera-Paredes, B.; Nikolov, S.; Jain, R.; Adler, J.; Back, T.; Petersen, S.; Reiman, D.; Clancy, E.; Zielinski, M.; Steinegger, M.; Pacholska, M.; Berghammer, T.; Bodenstein, S.; Silver, D.; Vinyals, O.; Senior, A. W.; Kavukcuoglu, K.; Kohli, P.; Hassabis, D. Highly Accurate Protein Structure Prediction with AlphaFold. *Nature 2021 596:7873* **2021**, *596* (7873), 583–589. https://doi.org/10.1038/s41586-021-03819-2.

(20) Long, F.; Nicholls, R. A.; Emsley, P.; Gražulis, S.; Merkys, A.; Vaitkus, A.; Murshudov, G. N. AceDRG: A Stereochemical Description Generator for Ligands. *Acta Crystallogr D Struct Biol* **2017**, *73* (Pt 2), 112–122. https://doi.org/10.1107/S2059798317000067.

(21) Murshudov, G. N.; Skubák, P.; Lebedev, A. A.; Pannu, N. S.; Steiner, R. A.; Nicholls, R. A.; Winn, M. D.; Long, F.; Vagin, A. A. REFMAC5 for the Refinement of Macromolecular Crystal Structures. *Acta Crystallogr D Biol Crystallogr* **2011**, *67* (Pt 4), 355–367. https://doi.org/10.1107/S0907444911001314.

(22) Emsley, P.; Lohkamp, B.; Scott, W. G.; Cowtan, K. Features and Development of Coot. *Acta Crystallogr D Biol Crystallogr* **2010**, *66* (Pt 4), 486–501. https://doi.org/10.1107/S0907444910007493.

(23) Joosten, R. P.; Long, F.; Murshudov, G. N.; Perrakis, A. The PDB_REDO Server for Macromolecular Structure Model Optimization. *IUCrJ* **2014**, *1* (Pt 4), 213. https://doi.org/10.1107/S2052252514009324.

(24) Afonine, P. V.; Grosse-Kunstleve, R. W.; Echols, N.; Headd, J. J.; Moriarty, N. W.; Mustyakimov, M.; Terwilliger, T. C.; Urzhumtsev, A.; Zwart, P. H.; Adams, P. D. Towards Automated

Crystallographic Structure Refinement with Phenix.Refine. *Acta Crystallogr D Biol Crystallogr* **2012**, *68* (Pt 4), 352–367. https://doi.org/10.1107/S0907444912001308.

(25) Liebschner, D.; Afonine, P. V.; Baker, M. L.; Bunkoczi, G.; Chen, V. B.; Croll, T. I.; Hintze, B.; Hung, L. W.; Jain, S.; McCoy, A. J.; Moriarty, N. W.; Oeffner, R. D.; Poon, B. K.; Prisant, M. G.; Read, R. J.; Richardson, J. S.; Richardson, D. C.; Sammito, M. D.; Sobolev, O. V.; Stockwell, D. H.; Terwilliger, T. C.; Urzhumtsev, A. G.; Videau, L. L.; Williams, C. J.; Adams, P. D. Macromolecular Structure Determination Using X-Rays, Neutrons and Electrons: Recent Developments in Phenix. *Acta Crystallogr D Struct Biol* **2019**, *75* (Pt 10), 861–877. https://doi.org/10.1107/S2059798319011471.

(26) Geoghegan, K. F.; Dixon, H. B. F.; Rosner, P. J.; Hoth, L. R.; Lanzetti, A. J.; Borzilleri, K. A.; Marr, E. S.; Pezzullo, L. H.; Martin, L. B.; Lemotte, P. K.; McColl, A. S.; Kamath, A. V.; Stroh, J. G. Spontaneous Alpha-N-6-Phosphogluconoylation of a "His Tag" in Escherichia Coli: The Cause of Extra Mass of 258 or 178 Da in Fusion Proteins. *Anal Biochem* **1999**, *267* (1), 169–184. https://doi.org/10.1006/ABIO.1998.2990.
